# Recent Progress in Catalyst Development of the Hydrogenolysis of Biomass-Based Glycerol into Propanediols—A Review

**DOI:** 10.3390/bioengineering10111264

**Published:** 2023-10-30

**Authors:** Lan Ma, Huimin Liu, Dehua He

**Affiliations:** 1 Institute of Chemical Defense, Beijing 102205, China; malan112@126.com; 2 School of Chemical and Environmental Engineering, Liaoning University of Technology, Jinzhou 121001, China; 3 Department of Chemistry, Tsinghua University, Beijing 100084, China

**Keywords:** glycerol, hydrogenolysis, 1,2-propanediol, 1,3-propanediol, catalyst

## Abstract

The use of biomass-based glycerol to produce chemicals with high added value is of great significance for solving the problem of glycerol surplus and thus reducing the production cost of biodiesel. The production of 1,2-propanediol (abbreviated as 1,2-PDO) and 1,3-propanediol (abbreviated as 1,3-PDO) via the hydrogenolysis of glycerol is one of the most representative and highest-potential processes for the comprehensive utilization of biomass-based glycerol. Glycerol hydrogenolysis may include several parallel and serial reactions (involving broken C–O and C–C bonds), and therefore, the catalyst is a key factor in improving the rate of glycerol hydrogenolysis and the selectivities of the target products. Over the past 20 years, glycerol hydrogenolysis has been extensively investigated, and until now, the developments of catalysts for glycerol hydrogenolysis have been active research topics. Non-precious metals, including Cu, Ni, and Co, and some precious metals (Ru, Pd, etc.) have been used as the active components of the catalysts for the hydrogenolysis of glycerol to 1,2-PDO, while precious metals such as Pt, Rh, Ru, Pd, and Ir have been used for the catalytic conversion of glycerol to 1,3-PDO. In this article, we focus on reviewing the research progress of the catalyst systems, including Cu-based catalysts and Pt-, Ru-, and Pd-based catalysts for the hydrogenolysis of glycerol to 1,2-PDO, as well as Pt-WO_x_-based and Ir-ReO_x_-based catalysts for the hydrogenolysis of glycerol to 1,3-PDO. The influence of the properties of active components and supports, the effects of promoters and additives, and the interaction and synergic effects between active component metals and supports are also examined.

## 1. Introduction

With the rapid depletion of fossil resources and the increasingly serious problems of CO_2_ emission and environmental pollution caused by the use of fossil resources, the development and utilization of renewable resources and clean energies, as well as the technologies for energy conservation and emission reduction, have been given great attention [1]. In the past 20 years, the utilization of biomass resources and biomass energy has been attributed great importance by many countries and regions, and related technologies have been rapidly developed [2,3]. Among them, biodiesel is a green energy, and the technology for its production has matured and been promoted and applied in many countries and regions [4,5]. However, with the large-scale production of biodiesel, glycerol, as a byproduct, will inevitably be produced in large quantities [6,7]. Nevertheless, the demand for conventional uses of glycerol (used in the production of skin care humectants, food sweet additives, antifreeze, nitroglycerin, solvents, etc.) is limited, which results in a glycerol surplus [8,9]. Therefore, it is important to develop downstream products with high added value of glycerol. Making glycerol with high added value is of great significance for solving the problem of excess glycerol and thereby reducing the production cost of biodiesel [10,11].

**Scheme 1:**
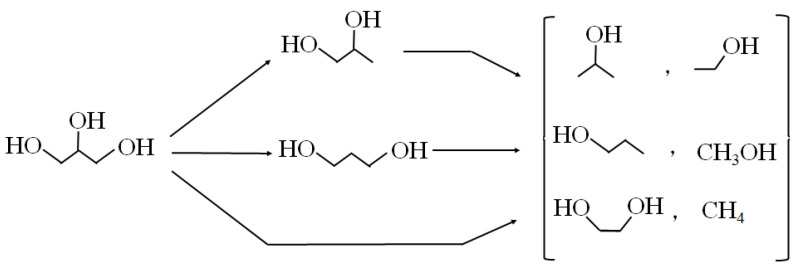
The reaction paths and products of glycerol hydrogenolysis.

There are many technical approaches (such as dehydration, hydrogenolysis, oxidation, carboxylation, esterification, etherification, pyrolysis gasification, etc.) to make glycerol with high added value and obtain downstream chemicals, such as binary alcohol, acrolein and acetol, dihydroxyacetone and glyceraldehydes, glyceric acid and hydroxypyruvic acid, glycerides, glycerol tert-butyl ether and polyglycerols, glycerol carbonate, etc. [12,13,14]. Among them, the production of 1,2-propanediol (1,2-PDO) and 1,3-propanediol (1,3-PDO) using glycerol hydrogenolysis is one of the most representative and highest-potential processes for the comprehensive utilization of biomass-based glycerol [15,16,17]. Of course, ethylene glycol, 1-propanol, 2-propanol, and ethanol can also be obtained through glycerol hydrogenolysis. The reaction paths and products of glycerol hydrogenolysis are shown in Scheme 1. In this paper, we focus on reviewing the recent progress of research on the hydrogenolysis of glycerol into 1,2-PDO and 1,3-PDO.

1,2-PDO and 1,3-PDO are important chemicals. In addition to being used as raw materials for polyester, plasticizer, and surfactant, 1,2-PDO is also widely used as a hygroscopic agent, food emulsifier, additive, antifreeze, lubricant, and solvent [6]. One of the most important uses of 1,3-PDO is as the raw material of high-performance polyester fiber, poly 1,3-propylene terephthalate (abbreviated as PTT). In addition, it is also used as a raw material for surfactants, emulsifiers, humectants, and medicine [18]. Currently, 1,3-PDO is much more expensive than 1,2-PDO on the market [15], and 1,2-PDO has more uses and greater market demand. Overall though, the market potential of these two types of propanediols is enormous.

In traditional industrial processes, 1,2-PDO is produced via the petrochemical route through the hydrolysis of propylene oxide, which is produced from propylene [6,18]. Similarly, the formation of 1,3-PDO is also based on the petrochemical approach in traditional industrial processes. Among the options, one process is first to obtain 3-hydroxypropion aldehyde from ethylene oxide through hydroformylation, and then to obtain 1,3-PDO through the hydrogenation of 3-hydroxypropion aldehyde; the other method is to obtain acrolein from propylene, produce 3-hydroxypropion aldehyde through a hydration reaction, and finally obtain 1,3-PDO by conducting hydrogenation [6,18]. From the perspective of reducing the use of fossil resources and comprehensively utilizing renewable resources, it is attractive to produce 1,2-PDO and 1,3-PDO through the hydrogenolysis of biomass-based glycerol, and this will be the main technological path for the production of these two kinds of chemicals in the future.

The reaction of glycerol and hydrogen at a certain temperature on a catalyst may include several parallel and serial reactions (involving broken C–C bonds and C–O bonds), and the products may be complicated. For instance, these hydrogenolysis products may include propanol, ethylene glycol, ethanol, methanol, and gas products (propane and methane). Therefore, regulating the reaction path and increasing the selectivity of the target product are crucial. From the perspective of industrial production, even if the products of glycerol hydrogenolysis are mainly 1,3-PDO and 1,2-PDO, it is desirable to maximize selectivity so that the selectivity of a single product (1,2-PDO or 1,3-PDO) is as high as possible, meaning the subsequent purification of products will be much simpler.

To improve the rate of glycerol hydrogenolysis and the selectivities of the target products, catalysts are the most important factor. In the past 20 years, glycerol hydrogenolysis has been extensively investigated. A large number of papers on the developments of catalysts used for glycerol hydrogenolysis, optimization of the reaction conditions, and reaction mechanisms have been published [19]. There have also been many review papers summarizing the progress of glycerol hydrogenolysis [6,15,16,17]. Furthermore, more than ten review articles have been published that specifically summarized the research progress on selectively converting glycerol to 1,3-PDO and 1,2-PDO through hydrogenolysis [15,16,17,19,20,21,22,23,24]. The development of catalysts for glycerol hydrogenolysis is still an active research topic. Currently, non-precious metals are mainly used as the active components of catalysts for the hydrogenolysis of glycerol to 1,2-PDO, while precious metals are adopted as the active components of catalysts in the selective transformation of glycerol to 1,3-PDO through hydrogenolysis.

In recent years, there have been many academic papers on glycerol hydrogenation to 1,2-PDO or 1,3-PDO published each year. Here, we summarize the latest progress of research on the development of catalysts for the hydrogenolysis of glycerol.

## 2. Catalysts for Selective Glycerol Hydrogenolysis to 1,2-PDO

**Figure 1:**
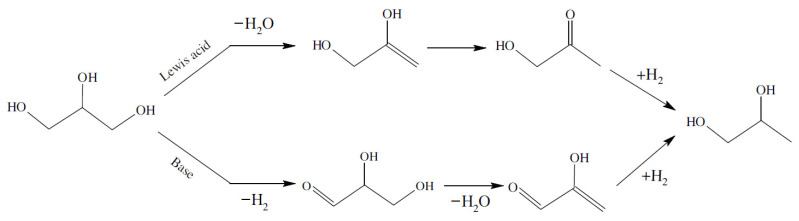
Reaction routes of glycerol hydrogenolysis to 1,2-PDO. Reproduced with permission from reference [16].

The hydrogenolysis of glycerol to 1,2-PDO generally involves dehydration and hydrogenation reactions or dehydrogenation–dehydration and hydrogenation steps (Figure 1) [16]. 1,2-PDO can be produced only when one of the primary -OH groups is hydrogenolyzed, and the selective catalytic activation of C-OH bonds is needed. In terms of the research on the developments of catalysts for the hydrogenolysis of glycerol to 1,2-PDO, various metals with hydrogenation activity have been screened and explored, which have been summarized in several review articles [16,17,19]. The non-precious metals used as active components on supported catalysts mainly include Cu, Ni, Co, etc., while the precious metals used as active components are mainly Ru, Pd, Pt, Rh, etc. The supports used include activated carbon, Al_2_O_3_, SiO_2_, ZnO, ZrO_2_, TiO_2_, CeO_2_, MgO, Cr_2_O_3_, La_2_O_3_, hydrotalcite, zeolites (MOR, ZSM-5, β, X, Y, etc.), and mixed oxides (such as ZnO-Al_2_O_3_, etc.) [16,17,19].

As glycerol hydrogenolysis involves dehydration and hydrogenation steps, catalysts with bifunctional active sites are often desirable. Usually, acid supports or base supports are used to construct dehydration active sites. γ-Al_2_O_3_, acidic zeolites (such as H-β, H-Y, H-MOR, H-ZSM-5, etc.), and other acidic metal oxides or mixed oxides are often used as acid supports, while MgO, La_2_O_3_, alkali-modified metal oxides, and hydrotalcites are often used as base supports [16,17,19]. On the other hand, acid or base compounds as additives may also be added into the reaction systems. It has been reported in the literature that ion-exchange resins (such as Amberlyst-15 and Nafion), heteropoly acids, hydrochloric acid, liquid H_2_SO_4_, etc., are often used as acidic additives, while LiOH, NaOH, KOH, Li_2_CO_3_, Na_2_CO_3_, K_2_CO_3_, etc., are selected as alkaline additives [16,17,19]. Precious metals have a relatively high ability to enter hydrogenation reactions and show high activity in glycerol hydrogenolysis, but they are expensive. For the hydrogenolysis of glycerol to 1,2-PDO, the results of catalysts screened by many researchers have shown that catalysts with Cu as the active component have relatively high activity and selectivity. Cu catalysts or Cu-based bimetallic catalysts modified by other metals (Ni, Co, Pd, Pt, Ru, etc.) are the most commonly employed catalysts in the hydrogenolysis of glycerol to 1,2-PDO [16,17,19,25].

In recent years, the catalysts for the hydrogenolysis of glycerol to 1,2-PDO reported in the literature have mainly been Cu-based catalysts, with a few noble metal (Pt, Ru, Pd)-based catalysts. In this section, the recent progress in the developments of catalysts for the selective hydrogenolysis of glycerol to 1,2-PDO is reviewed. According to the nature of the active components, the catalysts can be roughly divided into Cu-based catalysts and Pt-, Ru-, and Pd-based catalysts.

### 2.1. Cu-Based Catalysts

Regarding the research on converting glycerol via hydrogenolysis to 1,2-PDO, Cu-based catalysts have been widely employed and investigated in depth. Researchers have conducted extensive investigations on the selection of supports, the doping of additives, and the optimization of reaction conditions. Mane et al. [25] summarized some Cu-based catalysts used in the reaction of glycerol hydrogenolysis to 1,2-PDO and compared the catalytic performances of various Cu-based catalysts, including the types of catalysts, reaction conditions, conversions, and selectivities (Table 1). The detailed references related to Table 1 can be found in Mane’s review article [25].

In terms of Cu-based catalysts, the nature of Cu, the properties of the support, the synergic effect of Cu and the support, and the promoters play important roles in the catalytic performances of Cu-based catalysts in the selective hydrogenolysis of glycerol to 1,2-PDO [26,27,28,29].

#### 2.1.1. Influence of the Properties of the Cu Metal Component

It has been reported that the size of Cu [30] as well as the existence state of Cu active sites [31] can affect the performances of Cu-based catalysts in the selective hydrogenolysis of glycerol to 1,2-PDO. In different catalytic systems, sometimes controversial conclusions can be obtained.

In some catalytic systems, the sizes of Cu were crucial. For instance, Kolena et al. prepared several Cu-Al-Zn catalysts and evaluated their catalytic performances in the selective hydrogenolysis of glycerol to 1,2-PDO [30]. It was found that the activity of the catalysts correlated well with the size of the Cu nanoparticles, with small Cu nanoparticles favoring high catalytic activity (Table 2). In some other catalytic systems, the existence state of Cu active sites may have played the most important role. For instance, it was reported that the Cu_2_O phase was more active than the CuO phase in the selective hydrogenolysis of glycerol to 1,2-PDO [31]. Nikolaev et al. prepared a series of Cu/Al_2_O_3_ catalysts via a coprecipitation process from Cu(NO_3_)_2_ and Al(NO_3_)_3_ using NaOH and NH_4_OH as the precipitants. It was discovered that the selectivity to 1,2-PDO over the Cu/Al_2_O_3_ catalysts was 98%, and the activity depended on the properties of the catalysts. The characterization results suggested that the surface of Cu/Al_2_O_3_ was composed of Cu_2_O and CuO, with sizes in the range of 20 to 140 nm. Cu/Al_2_O_3_ catalysts with similar surface chemical compositions but different particle sizes of active phases exhibited similar specific activities, indicating that the hydrogenolysis reaction was not structure responsive when Cu/Al_2_O_3_ was used as the catalyst. Moreover, the decrease in the concentration of the Cu_2_O phase on the catalyst surface led to a decrease in the reaction rate, suggesting the higher activity of Cu_2_O than CuO in the selective hydrogenolysis of glycerol [31].

#### 2.1.2. Effects of Support Properties

Al_2_O_3_, MgO, dolomite, and CuB_2_O_4_ spinel are generally used as supports of Cu-based catalysts in the selective hydrogenolysis of glycerol to 1,2-PDO. In this subsection, based on the properties of the supports, we divide the Cu-based catalysts into catalysts with supports that have acid sites, catalysts with supports that have base sites or simultaneously contain acid–base sites, and catalysts with supports that have both acid sites and redox properties, and we review the research progress here.

**Figure 2:**
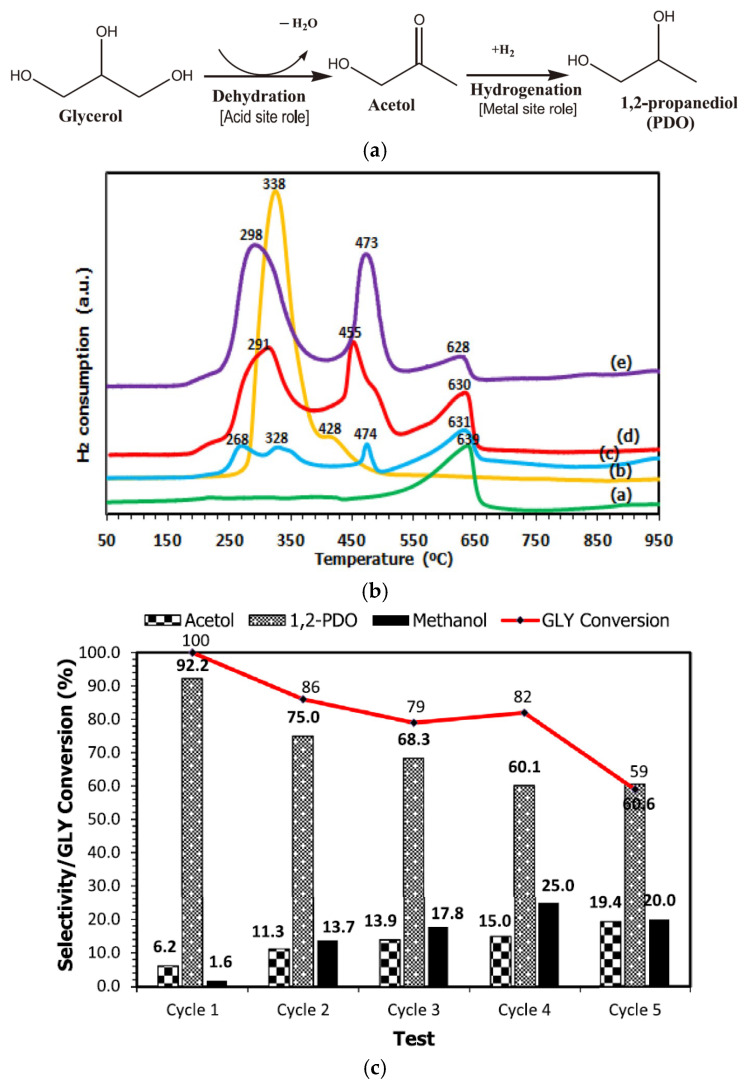
(**a**) Reaction pathway of glycerol hydrogenolysis to 1,2-PDO, (**b**) H_2_-TPR of a: dolomite, b: copper oxide, c: 10% Cu/dolomite, d: 20% Cu/dolomite, e: 30% Cu/dolomite, and (**c**) stability test of 20% Cu/dolomite in selective glycerol hydrogenolysis to 1,2-PDO. Reproduced with permission from reference [37].

(1) Catalysts with supports that have acid sites. The selective hydrogenolysis of glycerol to 1,2-PDO generally proceeds via two steps. Glycerol is initially dehydrated to form acetol and then acetol is hydrogenated to 1,2-PDO. The dehydration reaction occurs over acid and/or base sites, while the hydrogenation reaction requires active metallic sites (Figure 2a). Therefore, Cu/acidic support catalysts are expected to be active in the selective hydrogenolysis of glycerol to 1,2-PDO.

A_2_O_3_ is the most widely investigated acidic support in this research area [32,33]. For example, over the precursor of the Raney-Cu/Al_2_O_3_ catalyst, CuAl_2_ was the main crystal phase after calcining it at 850 °C in air. A portion of CuAl_2_ was oxidized to α-Al_2_O_3_ while the remaining CuAl_2_ was converted to active skeletal Cu after leaching. On the as-prepared Raney-Cu/Al_2_O_3_ catalyst, a conversion of glycerol of 30.9% and a selectivity towards 1,2-PDO of 91.4% were realized under the conditions of a reaction temperature of 215 °C, H_2_ pressure of 3 MPa, and LHSV of 1.0 h^−1^ [34]. A kinetic study suggested that over a 60 wt% Cu/Al_2_O_3_ catalyst, the dehydration of glycerol to acetol was the rate-limiting step for the production of 1,2-PDO, with zero and first orders with respect to hydrogen and glycerol, respectively [35]. Modifying Cu/Al_2_O_3_ catalysts with B_2_O_3_ could enhance their acidity, accelerate the rate-determining dehydration step, and boost the catalytic activities of the catalysts [36].

The high acidity of dolomite makes it another promising support for Cu-based catalysts for the selective hydrogenolysis of glycerol to 1,2-PDO. For instance, Ramli et al. [37] synthesized a series of Cu/dolomite catalysts, where metallic Cu species were highly dispersed on the surfaces of dolomite particles. It was discovered that doping Cu onto dolomite increased its acid content and strength. The loading of Cu onto dolomite improved the redox properties of the Cu/dolomite catalysts because the reduction temperatures of the catalysts were lower than those of pure CuO and dolomite support (Figure 2b). Notably, the optimized 20 wt% Cu/dolomite catalyst gave 100% glycerol conversion and 92% 1,2-PDO selectivity under the conditions of 2 MPa H_2_, 180 °C, and 6 h reaction time, although its stability needed further improvement (Figure 2c) [37].

**Figure 3:**
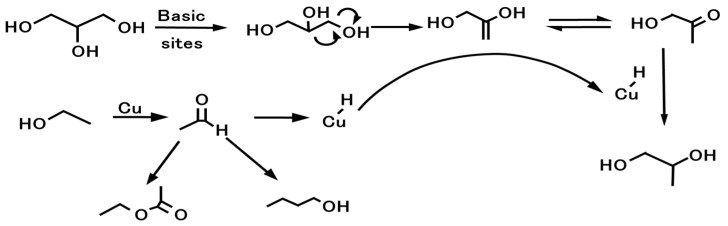
Proposed reaction mechanism of selective glycerol hydrogenolysis to 1,2-PDO over Cu-Al-Zn catalysts. Reproduced with permission from reference [39].

(2) Catalysts with supports that have base sites or simultaneously contain acid–base sites. As both acid and base sites can drive the glycerol to acetol dehydration process, supports with both acidic and basic properties are also promising for the selective hydrogenolysis of glycerol to 1,2-PDO. For example, Biswas et al. [38] demonstrated that Cu/MgO was highly active, selective, and stable for the hydrogenolysis of glycerol to 1,2-PDO. Notably, 10 wt% Cu/MgO exhibited a 100% conversion of glycerol and a 95.5% yield towards 1,2-PDO at a WHSV of 1.2 h^−1^, 220 °C, and 0.75 MPa pressure. The presence of bifunctional acidic–basic sites, small Cu particle size, and the synergetic interaction between Cu nanoparticles and MgO support contributed to the superior catalytic performances of Cu/MgO, which promoted the selective hydrogenolysis of C−O bonds for high activity and limited the cleavage of C−C bonds to improve selectivity [38]. Kumar et al. [39] applied Cu-Al-Zn catalysts in the conversion of glycerol to 1,2-PDO, with the optimized Cu-Al-Zn catalysts recording a glycerol conversion of 43% and a selectivity towards 1,2-PDO of 69% under the conditions of 200 °C, 30 bar N_2_, 0.088 mol ethanol, 0.022 mol glycerol, and 0.25 g catalyst. Characterization revealed that there were both base and acid sites over the Cu-Al-Zn catalysts. The acid and base sites responded to catalytic transfer hydrogenation while Cu impacted the C-O/C–C cleavage of glycerol (Figure 3) [39].

(3) Catalysts with supports of acidic and redox properties. Supports with redox properties can generally promote the reduction of Cu species at low temperatures and enhance the interactions between Cu and the support. In this regard, materials with both acidic and redox properties are promising as supports for Cu-based catalysts for the selective hydrogenolysis of glycerol to 1,2-PDO. Cu/ZrO_2_ is a typical example that falls into this category. Landi et al. [40] reported that a 1,2-PDO yield of 75% could be obtained over Cu/ZrO_2_ catalyst, with the good catalytic performances of Cu/ZrO_2_ being assigned to its redox and acidic properties. The appropriate amount of Cu_2_O and the weak acidic sites on ZrO_2_ were essential for the selective hydrogenolysis pathway, which was favorable for avoiding the excessive hydrogenolysis of 1,2-PDO to 1-propanol and enhancing the dehydration step of glycerol to hydroxyl acetone, and subsequently to 1,2-PDO via the hydrogenation of the latter.

#### 2.1.3. Cu Metal and Support Synergic Effect

The synergic effect between the metal and support occasionally endows the catalysts with unique properties and then contributes to the enhancement of their performances in the selective hydrogenolysis of glycerol to 1,2-PDO. Biswas et al.’s study is a typical example. The authors prepared a CuMgAl-O catalyst from LDH (layered double hydroxides) via the hydrolysis method and further incorporated Zn into CuMgAl-O. Compared with CuMgAl-O oxide, the CuZnMgAl-O catalyst exhibited higher glycerol conversion, slightly decreased selectivity to 1,2-PDO, increased selectivity to ethylene glycol, and nearly constant selectivities to other products. Over the optimized CuZnMgAl-O catalyst, 98.4% glycerol conversion and 94.3% 1,2-PDO selectivity could be achieved under the optimized reaction conditions (1 g of NaOH, 210 °C, 4.5 MPa, and 12 h). Catalyst characterizations suggested that ZnO facilitated catalyst reduction at low temperatures due to the hydrogen spillover effect of ZnO to Cu. The synergic effect between Cu, ZnO, and the support enhanced the catalytic activity [28].

#### 2.1.4. Effects of Promoters

The modification of Cu-based catalysts by using suitable additives can further improve their catalytic performances in the selective hydrogenolysis of glycerol to 1,2-PDO [41,42]. For example, Hwang et al. [43] modified Cu/SiO_2_ with Ni and constructed a Ni-Cu/SiO_2_ nanocomposite catalyst. It was discovered that adding a small amount (3 wt%) of Ni to Cu-SiO_2_ could decrease the particle size of Cu, which is beneficial for attaining good catalytic performance in the selective hydrogenolysis of glycerol. Notably, the 3 wt% Ni-Cu/SiO_2_ catalyst showed high activity for the hydrogenolysis of glycerol to 1,2-PDO (100% glycerol conversion and 92% 1,2-PDO selectivity) under the optimized conditions (220 °C, 30 bar, and 0.5 h^−1^ WHSV), which was much higher than that of the unmodified catalyst [43].

### 2.2. Pt-, Ru-, and Pd-Based Catalysts

Pt-, Ru-, and Pd-based catalysts have also been investigated in the selective hydrogenolysis of glycerol to 1,2-PDO. In this subsection, we will describe the research progress of Pt-, Ru-, and Pd-based catalysts [44,45,46].

#### 2.2.1. Pt-Based Catalysts

**Figure 4:**
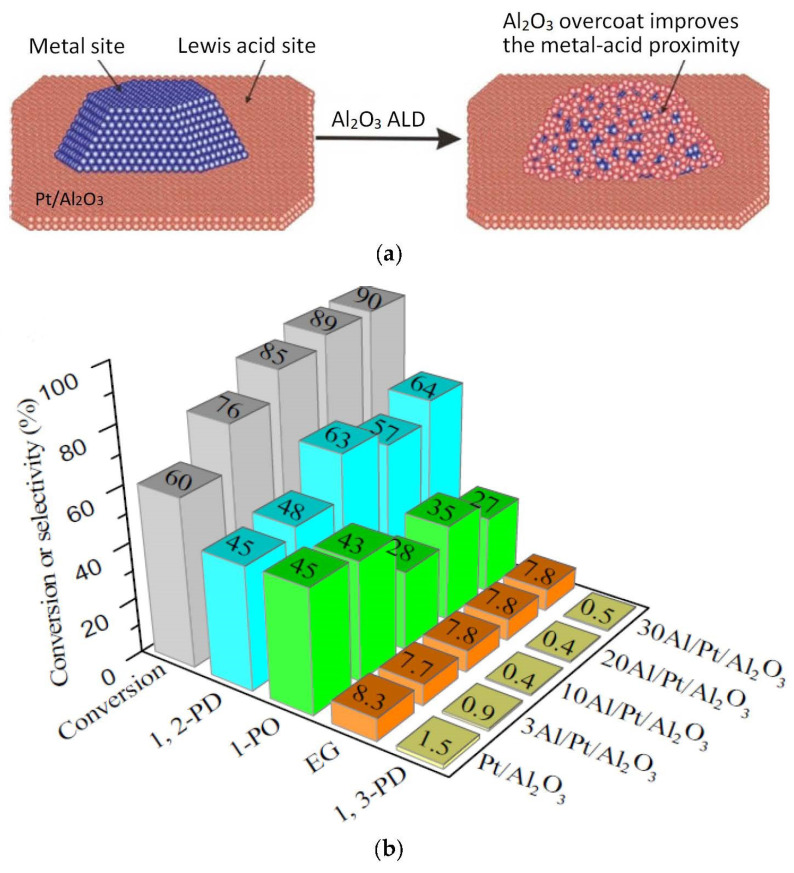
(**a**) Schematic illustration of the preparation of Al_2_O_3_-overcoated Pt/Al_2_O_3_, and (**b**) catalytic performances of Pt/Al_2_O_3_ and Al_2_O_3_-overcoated Pt/Al_2_O_3_ in selective glycerol hydrogenolysis to 1,2-PDO. Reproduced with permission from reference [44].

Pt/Al_2_O_3_ is a bifunctional catalyst that contains both metallic and acid sites, which makes it applicable for the selective hydrogenolysis of glycerol to 1,2-PDO. The distance between the metallic active site and acid sites is crucial for its catalytic performances. Lu et al. [44] coated porous Al_2_O_3_ onto the surface of Pt/Al_2_O_3_ to improve the proximity between Pt and Al_2_O_3_ (Figure 4a). Characterization revealed that the Al_2_O_3_ overcoat did not change the catalyst acidity considerably but enlarged the area of the Pt-Al_2_O_3_ interface. In the selective hydrogenolysis of glycerol to 1,2-PDO, the Al_2_O_3_-overcoated Pt/Al_2_O_3_ exhibited an approximately 2.8-fold activity and high selectivity to 1,2-PDO at high conversions (Figure 4b). Controlled experiments indicated that the enhanced Pt-Al_2_O_3_ interface accelerated the glycerol dehydration step and improved the catalytic activity [44].

Bimetallic catalysts generally exhibit better properties and performances than their corresponding monometallic catalysts. Based on this, Pt-based bimetallic catalysts, such as Cu-Pt/SiO_2_ [45], Au-Pt/TiO_2_ [46], and Pt-In alloy [47], have been developed with the aim of improving their performances in the selective hydrogenolysis of glycerol to 1,2-PDO. Here, Pt-In alloy is taken as an example for illustration. In the literature, the electronic and geometric structure of the Pt-In alloy could be tuned by varying the ratio between Pt and In. The optimized Pt-In alloy exhibited 99.8% glycerol conversion, 91.1% 1,2-PDO selectivity, and 222 h^−1^ TOF (turnover frequency) in the selective hydrogenolysis of glycerol to 1,2-PDO, which were much higher than those of the non-alloyed Pt catalyst. The structure of the Pt-In alloy was conducive to the improvement of catalytic performances, where Pt^δ−^ on the interface of Pt-In served as an active center for improving the activation of glycerol, whereas the cleavage of the C−C bond was suppressed by the isolated Pt sites. Thus, the alloyed Pt-In changed the catalytic active sites, promoted the RDS, and improved the catalytic activity [47].

#### 2.2.2. Ru-Based Catalysts

Monometallic Ru-based catalysts, such as Ru/K-OMS-2 [48] and Ru/La-ZrO_2_ [49], could selectively hydrogenolyze glycerol to 1,2-PDO. For instance, Chilukuri et al. [48] prepared a series of Ru/K-OMS-2 catalysts with different Ru contents (1 wt%, 2 wt%, 3 wt%, and 5 wt%) and evaluated their performances in glycerol hydrogenolysis. The results revealed that 1 wt% Ru/K-OMS-2 afforded a higher yield of 1,2-PDO than the other catalysts. The better dispersion of Ru, higher active metal surface area, and suitable basic strength accounted for the enhanced catalytic performances [48].

Promoting monometallic Ru-based catalysts with acidic WO_x_ could further improve their catalytic performances [50]. The Ru-WO_x_/C catalyst was highly efficient for glycerol hydrogenolysis to 1,2-PDO, with the optimal catalyst giving 99% glycerol conversion and 98% 1,2-PDO selectivity. Characterization suggested that on Ru-WO_x_/C, Ru and W were uniformly supported on the surface of active carbon, with Ru sizes smaller than 2 nm and WO_x_ clusters smaller than 100 nm. The synergic effect between Ru and WO_x_ promoted the reaction, where the acid sites on WO_x_ allowed for the adsorption and activation of glycerol to form prop-2-ene-1,2-diol via the dehydration path, while Ru metallic sites were in response to the hydrogenation of prop-2-ene-1,2-diol to 1,2-PDO, via H spillover onto the surface [50].

Ru-based bimetallic catalysts, Ru-Co/ZrO_2_ [51], Ru-Cu/CNT [52], etc., have also been developed for the selective hydrogenolysis of glycerol to 1,2-PDO. Adding Cu to Ru/CNT could significantly enhance the reducibility and modify the properties of Ru-Cu species. The chemical composition of the bimetallic Ru-Cu catalyst together with the interaction between Cu and Ru promoted C–O bond cleavage instead of a C–C bond, which was conducive to the superior selectivity towards 1,2-PDO (up to 93.4%) for Ru-Cu/CNT [52].

#### 2.2.3. Pd-Based Catalysts

Pd nanoparticles on acidic supports can carry out the selective hydrogenolysis of glycerol to 1,2-PDO [53,54]. For instance, MOF-808-SO_4_-Pd [54], a catalyst with Pd nanoparticles on sulfate-functionalized metal–organic frameworks, was effective for glycerol hydrogenolysis, giving relatively high activity (93.9% 1,2-PDO selectivity, reaction rate = 22.4 mmol g_Pd_^−1^ h^−1^). The synergistic effects between MOF and Pd were responsible for the good catalytic performances, in which the sulfate groups in MOF drove the dehydration of glycerol, while Pd nanoparticles subsequently hydrogenated the intermediates to 1,2-PDO [54].

Pd-based bimetallic catalysts are generally more active than monometallic catalysts in the selective hydrogenolysis of glycerol to 1,2-PDO [55,56]. For example, the CuPd/TiO_2_-Na catalyst [55] could afford a TOF (based on Cu + Pd sites) of 0.14 s^−1^ and a selectivity to 1,2-PDO of 85% under the conditions of 220 °C and 0.7 MPa H_2_, which were much higher than those of the corresponding monometallic catalysts. Characterization suggested that a CuPd alloy was formed over the CuPd/TiO_2_-Na catalyst, with the average diameter of the CuPd alloy being smaller than that of the Cu or Pd nanoparticles of the monometallic catalysts. In addition, in the glycerol hydrogenolysis reaction, Pd promoted the activation of glycerol while Cu improved the subsequent hydrogenation step. As a result, high activity and 1,2-PDO selectivity could be realized over CuPd/TiO_2_-Na [55].

## 3. Selective Glycerol Hydrogenolysis to 1,3-PDO over Pt-Based and Ir-Based Catalysts

**Figure 5:**
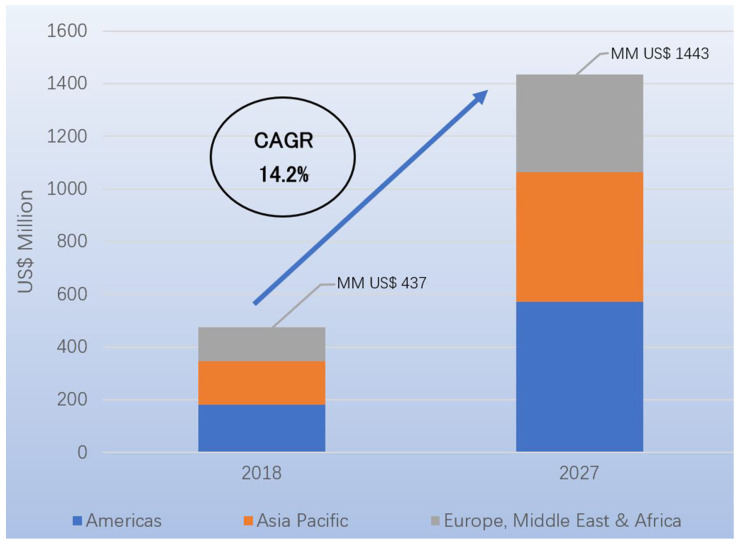
Global market of 1,3-PDO by region. Reproduced with permission from reference [15].

As mentioned above, 1,3-PDO can be used as the key raw material of high-performance polyester fiber (PTT), and it has high added value and growing market demand. Ruy et al. reported that the market for 1,3-PDO is optimistically expected to be USD 1443 million in 2027 (Figure 5) [15].

**Figure 6:**
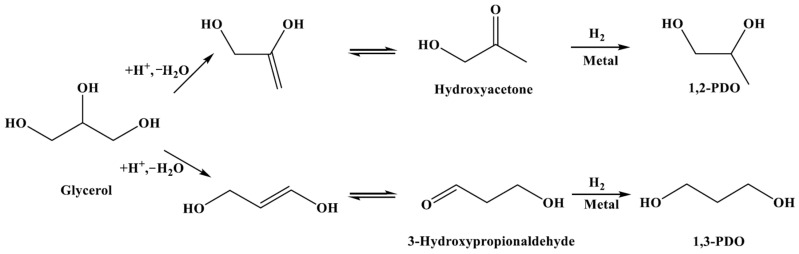
Glycerol hydrogenolysis forming propanediols (PDOs). Reproduced with permission from reference [24].

For the industrial production of 1,3-PDO from biodiesel-based glycerol, the high selectivity of the target product is still the key parameter. For selective glycerol hydrogenolysis, 1,3-PDO is generated only when the secondary -OH group is hydrogenolyzed (Figure 6) [24]. Due to the existence of two primary -OH groups that constitute a steric hindrance, the C–O bond activation of the secondary -OH group is hindered. In addition, the properties of the active sites of bifunctional catalysts are also related to the selectivity of 1,3-PDO. Although dehydration and hydrogenation reactions are also involved in the hydrogenolysis of glycerol to produce 1,3-PDO, the requirements for the properties of dehydration active sites and hydrogenation active sites on the catalysts are not the same as those for the production of 1,2-PDO. It has been reported in the literature that the Bronsted acid sites on the surface of bifunctional catalysts and the synergistic interaction between Bronsted acid sites and metal active sites are important factors for the generation of 1,3-PDO [15,20,22,23,57,58,59,60]. Some metal oxides, such as WO_x_, MoO_x_, and ReO_x_, could provide Bronsted acid sites (in situ generated) in hydrogenolysis [15,20,22,23,57,58,59,60]. Pt-metal-based catalysts modified with WO_x_, and Ir (as well as Rh or Ru)-metal-based catalysts modified with ReO_x_, such as Pt-WO_x_, Ir-ReO_x_, Rh-ReO_x_, or Ru-ReO_x_, can significantly improve the catalytic activities and the selectivity of 1,3-PDO [20,61,62,63,64,65,66,67,68].

Several review articles have also summarized and commented on catalysts that were employed in the hydrogenolysis of glycerol to 1,3-PDO [15,16,17,20,21,22,23,24,57,58,59]. In recent years, researchers have mainly focused on the modification of Pt-based and Ir-based catalysts to further increase the catalytic activity and the selectivity of 1,3-PDO. In this section, the recent research progress on Pt-WO_x_ catalysts and Ir-ReO_x_ catalysts for the selective hydrogenolysis of glycerol to produce 1,3-PDO is reviewed [69,70,71,72,73,74,75,76,77,78,79,80,81,82,83,84,85,86,87,88,89,90,91,92,93,94,95,96,97,98,99,100,101,102,103,104,105,106,107,108,109].

### 3.1. Pt-Based Catalysts

For the catalytic hydrogenolysis of glycerol to 1,3-PDO over Pt-WO_x_-based catalysts, glycerol is first dehydrated to 3-hydroxypropanal on acidic sites, and then the latter is hydrogenated on metal sites to form 1,3-PDO [20,21,22,23,69,70]. Pt-based catalysts with WO_x_ have shown high activity and selectivity to 1,3-PDO, and the catalysts have been thoroughly investigated [15,20,21,22,23,24,69,70,71,72,73,74,75,76,77,78,79,80,81,82,83,84,85,86,87,88,89,90,91,92,93,94,95,96,97,98,99].

#### 3.1.1. WO_x_ Species and Supports

In Pt-WO_x_-catalyzed glycerol hydrogenolysis, Pt is used to activate hydrogen molecules, while WO_x_ is usually employed to provide acid sites. Pt particle size and the surface area of WO_x_ (Bronsted acid) play important roles in providing a large number of active sites. Wang and Zhang’s group [69] prepared a single/pseudo-single-atom Pt catalyst using mesoporous WO_x_ alone as the support, thereby avoiding the interference caused by the use of other cosupports.

**Figure 7:**
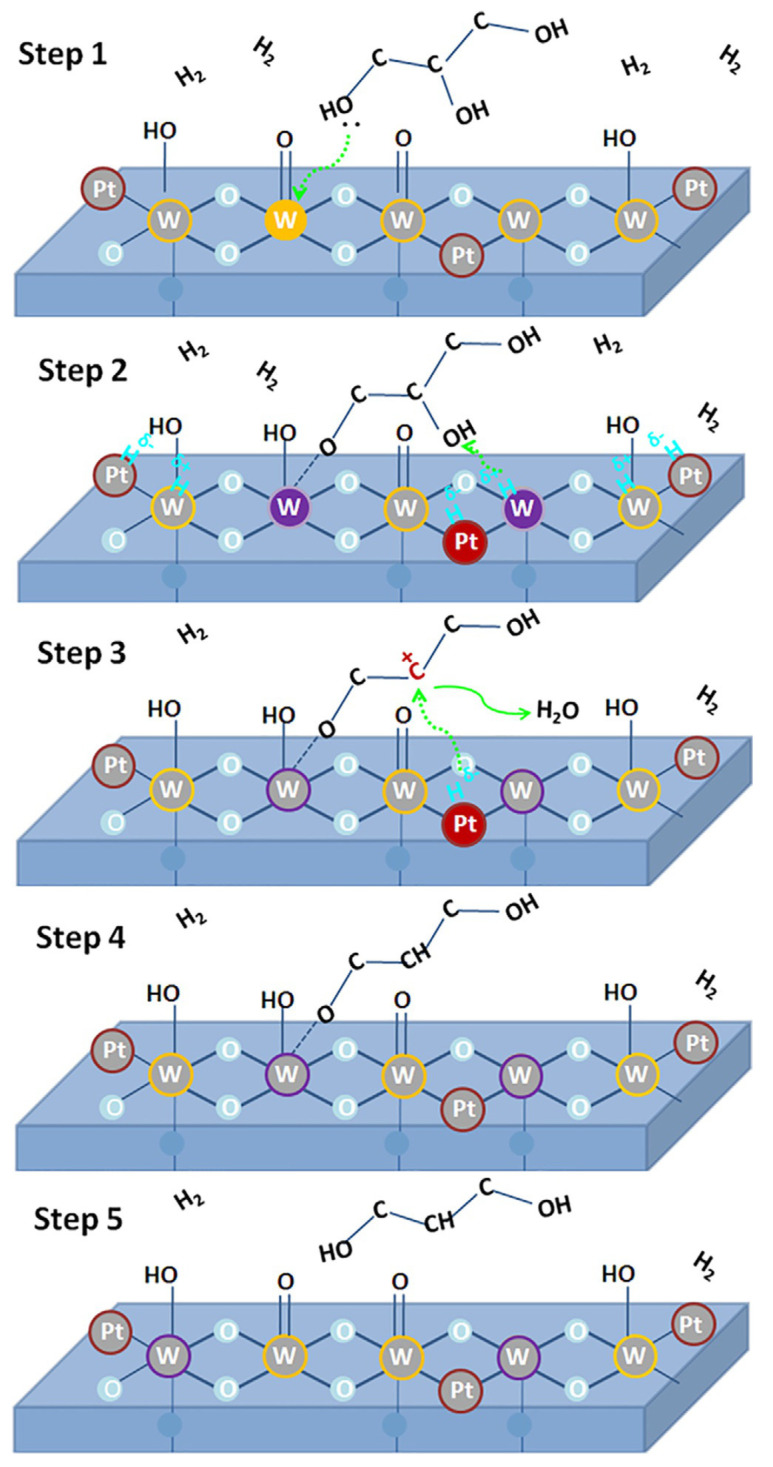
Proposed reaction scheme for glycerol hydrogenolysis to 1,3-PD over Pt/WO_x_. Reproduced with permission from reference [69].

Pt metal could be well dispersed on the mesoporous WO_x_, which had a large specific surface area, and the isolated Pt could be stabilized by the oxygen vacancies of WO_x_ [69]. The Pt/meso-WO_x_ catalyst showed excellent activity for the hydrogenolysis of glycerol and a relatively high yield of 1,3-PDO under 1 MPa H_2_ pressure and very high space-time. The authors thought that WO_x_ provided Bronsted acid sites, while the structure of isolated Pt boosted the dissociation of H_2_, and the synergistic effect of WO_x_ and Pt promoted dehydration and hydrogenation reactions. They finally concluded that the hetero dissociation of hydrogen molecules at the interfaces between Pt and WO_x_ was beneficial for the formation of 1,3-PDO, while the interaction between glycerol and the Bronsted acid sites of WO_x_ favored the formation of the intermediate of secondary carbon-cation. The authors also proposed a reaction scheme (Figure 7) for the hydrogenolysis of glycerol to 1,3-PDO over Pt/WO_x_, which focused on the bond formation of glycerol-WO_x_ and the interaction of glycerol and WO_x_ and emphasized the roles of Bronsted acid sites catalyzing the dehydration of glycerol and the interface between Pt and WO_x_ [69].

If only WO_3_ oxide is used as the support, it is difficult to highly disperse WO_3_ and Pt metal. Therefore, most researchers usually use thermally stable materials with large specific surface areas as the support of Pt and WO_x_ components, so that Pt and WO_x_ can be well dispersed on the surface of the support. The different supports used in Pt-WO_x_-based catalysts include various metal oxides and some zeolites, such as ZrO_2_ [71,72,73,74,75], Al_2_O_3_ [76,77,78], SiO_2_ or SBA-15 and Si-foams [70,79,80,81,82,83], SAPO-34 and H-MOR zeolites [84,85], Ta_2_O_5_ [86], and TiO_2_ [87].

**Figure 8:**
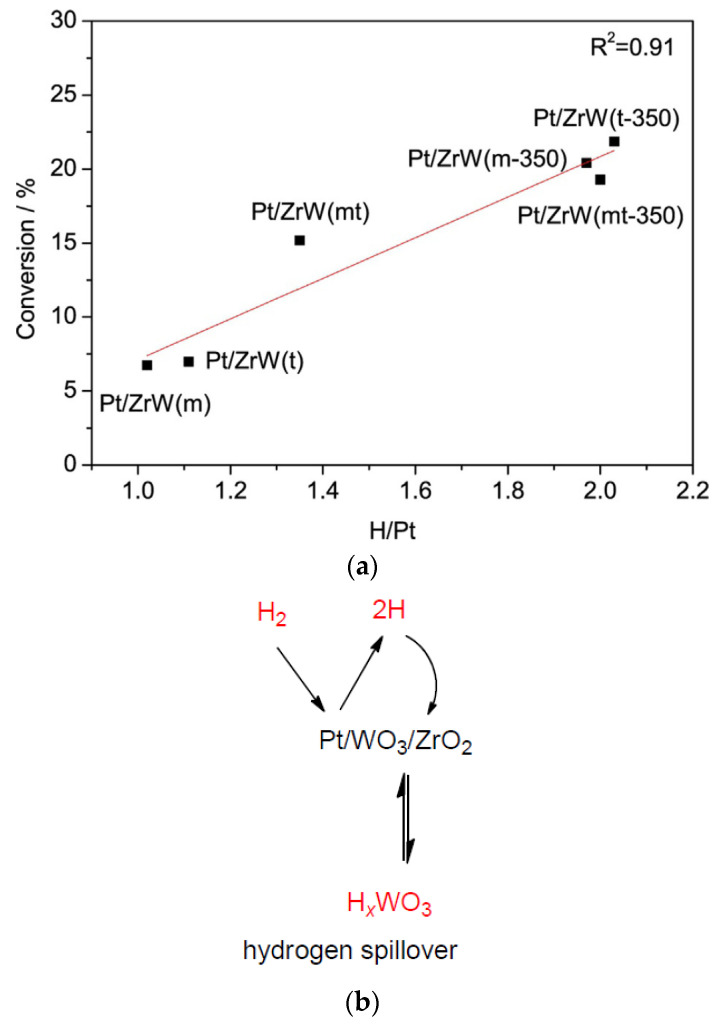
(**a**) Correlation between H/Pt and glycerol conversion (reproduced with permission from [71]), and (**b**) generation of H*x*WO_3_ via hydrogen spillover (reproduced with permission from reference [72]).

In studies of glycerol hydrogenolysis to 1,3-PD over Pt/WO_x_, ZrO_2_ is one of the commonly used supports for loading WO_x_ and Pt [22,23,61,62]. In the Pt/WO_x_/ZrO_2_ catalyst system, ZrO_2_ is not simply a support, and the properties of ZrO_2_ may also affect the catalytic performances of Pt/WO_x_/ZrO_2_ [71,72,73,74,75]. Ma’s group employed nano ZrO_2_ prepared using a solvothermal method as the support for loading WO_3_ and Pt metal, and investigated the effect of a tetragonal/monoclinic ZrO_2_ support and the influence of hydrogen spillover on the catalytic performances of Pt/WO_3_/ZrO_2_ [71]. They tuned the H-spillover capacities by changing ZrO_2_ crystalline structures (composition ratio of tetragonal phase to monoclinic phase) and the conditions of pretreatment of the Pt precursor, and found that there was a linear relationship between the H/Pt ratio and glycerol conversion (Figure 8a), showing the important role of hydrogen spillover for glycerol hydrogenolysis on these WO_3_-enriched Pt/WO_3_/ZrO_2_ nanocatalysts. Fan et al. also proposed a model of hydrogen spillover on the surface of Pt/WO_3_/ZrO_2_ (Figure 8b) [72]. For the Pt/WO_3_/ZrO_2_ catalyst, it was also considered that the active sites of Pt metal were responsible for the hydrogen activation and spillover step, and the WO_x_ species also had H-spillover capability for proton transfer from the dissociation of hydrogen molecules [73]. In addition, the investigation results of Ma’s group indicated that the dissociation of hydrogen molecules occurred preferentially on Pt(111) terraces [71]. However, Ma et al. considered that the strength of acids and the total number of acid sites on the Pt/WO_3_/ZrO_2_ catalyst showed no influence on glycerol conversion [71], and further investigations are needed to reveal the detailed catalytic mechanism of Pt/WO_3_/ZrO_2_.

**Figure 9:**
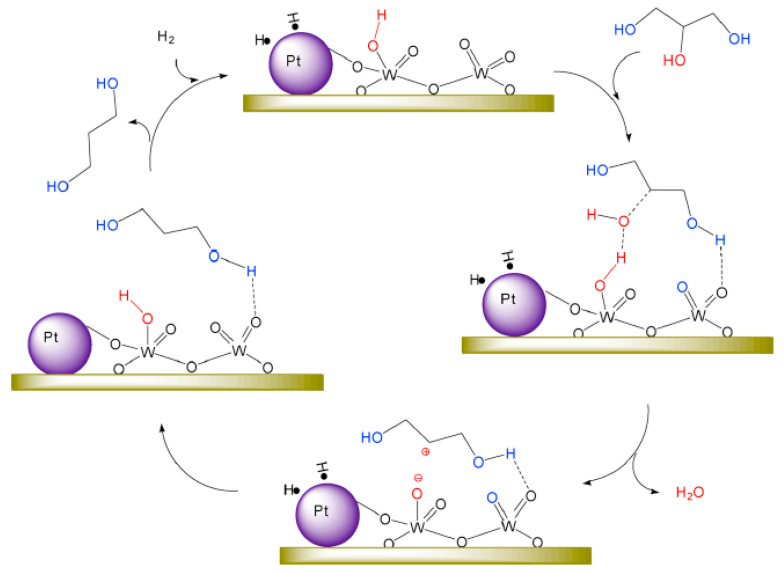
Reaction mechanism of glycerol hydrogenolysis to 1,3-PDO on super acidic Pt-(WO_x_)_n_-H structure. Reproduced with permission from reference [75].

Zhou et al. further studied the structures of the acid sites of Pt/WO_x_/ZrO_2_ catalyst systems and the catalytic working mechanism in the hydrogenolysis of glycerol to 1,3-PDO [75]. They prepared Pt/WO_x_/ZrO_2_ catalysts containing various amounts of WO_x_, adjusted the domain size of surface WO_x_ by changing WO_x_ loading or by doping Mn into WO_x_/ZrO_2_, and characterized the structure and acid properties of WO_x_ species. The results indicated that Pt particles and polymerized WO_x_ of medium size interacted, resulting in the generation of super strong Bronsted acid sites. The hydrogenolysis of glycerol was a structural-sensitive reaction to the domain size of WO_x_, and polymerized WO_x_ with a medium size was conducive to the formation of 1,3-PDO. They proposed a catalytic working mechanism of Pt/WO_x_/ZrO_2_ with a super acidic Pt-(WO_x_)_n_-H structure (combined super strong Bronsted acid site and Pt metal site) in the reaction of glycerol hydrogenolysis to 1,3-PDO (Figure 9) [75].

**Figure 10:**
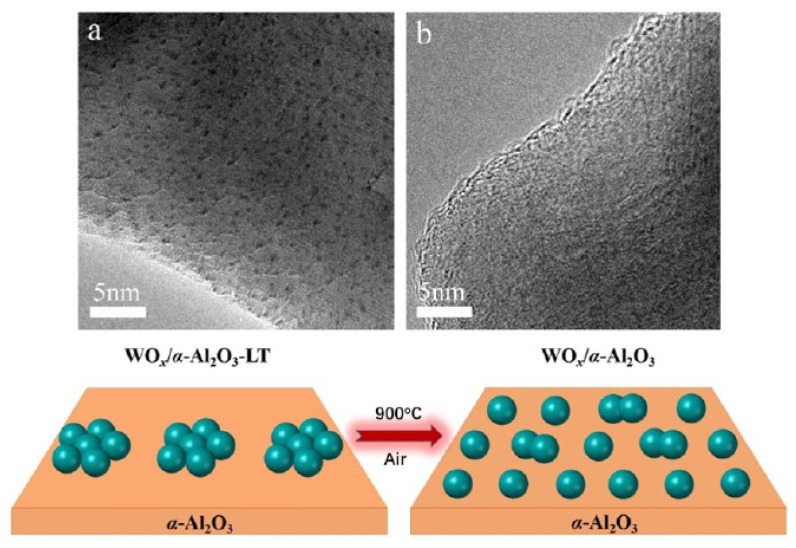
Representative TEM bright-field images and the corresponding schematic diagram of (**a**) the WO_x_/α-Al_2_O_3_-LT and (**b**) the WO_x_/α-Al_2_O_3_ samples, highlighting the difference in the dispersion of WO_x_ on the α-Al_2_O_3_ surface. Reproduced with permission from reference [78].

Al_2_O_3_ is also widely used as a support for Pt-WO_x_ [22,23,76,77,78]. Edake et al. prepared a Pt/WO_3_/Al_2_O_3_ catalyst, employed it in the gas-phase hydrogenolysis of glycerol to 1,3-PDO in a fluidized bed reactor (ambient pressure, 260 °C), and obtained a 14% yield of 1,3-PDO [76]. On the other hand, Wang and Zhang’s group employed a series of Pt/WO_x_/Al_2_O_3_ catalysts in the gas-phase hydrogenolysis of glycerol to 1,3-PDO in a fixed-bed reactor [77]. They changed the Pt/W atomic ratio in xPt/yWO_3_/Al_2_O_3_ catalysts and found that both Pt and WO_3_ had significant influences on the 1,3-PDO yield and that the number of Pt-WO_x_ interfacial sites reached a maximum in the range of 7~15 wt% W, where WO_x_ had a medium domain size. They also suggested that the strong Bronsted acid sites were generated in situ through the dissociation of hydrogen molecules as well as H spillover, and the hydrogenolysis of glycerol occurred at the Pt-WO_x_ interface. Furthermore, Zhao et al. [78] first prepared highly dispersed WO_x_ species on α-Al_2_O_3_ (Figure 10) and then fabricated a series of Pt/WO_x_/*α*-Al_2_O_3_ catalysts with different amounts of Pt-WO_x_ via the impregnation method and adjusting the Pt loading. The investigation results showed that the activated Al_2_O_3_ surface could enhance the interaction between the W species and the support, which was beneficial for the dispersion of WO_x_ species and promoted the formation of Pt-WO_x_ interfaces. They suggested that the enhanced catalytic performances of Pt/WO_x_/α-Al_2_O_3_ in the hydrogenolysis of glycerol to 1,3-PDO came from the synergistic effect between platinum species and the isolated WO_x_ species, which was conducive to the in situ generation of Bronsted acid sites during the hydrogenolysis of glycerol.

SiO_2_ has a large specific surface area and high hydrothermal stability, and it has been used as a support for Pt-WO_x_ [20,22,23,70,79,80,81,82,83]. Shi et al. [79] investigated the influence of doping WO_x_ into Pt/SiO_2_, and they found that there was a strong interaction between Pt metal and WO_x_ species (polytungstate being the predominant species). Additionally, doping WO_x_ boosted the acidity of Pt/SiO_2_ and enhanced the dispersion of Pt metal on WO_x_/SiO_2_, which markedly improved the conversion of glycerol and the selectivity of 1,3-PDO. SBA-15 is a kind of SiO_2_ with a well-ordered mesoporous structure, and some Pt-WO_x_ catalysts with W-SBA-15 or (W+Al)-SBA-15 as the support have also been examined for the hydrogenolysis of glycerol to 1,3-PDO [70,80].

**Figure 11:**
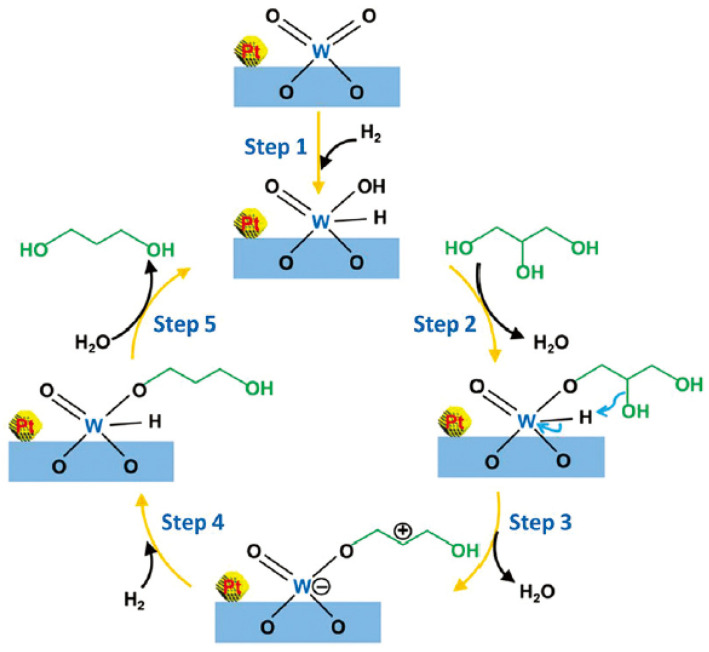
Reaction mechanism for the glycerol hydrogenolysis to 1,3-PDO over the Pt/W-SBA-15 catalysts. Reproduced with permission from reference [70].

Qiao’s group [70] prepared WO_x_-SBA-15 supports with extremely low amounts of tungsten (W/Si atomic ratios ≤ 1/80) via a sol–gel hydrothermal process, obtained a series of Pt/WO_x_-SBA-15 catalysts through an impregnation process, and found that Pt/WO_x_-SBA-15 with a low W/Si ratio (1/640) showed good catalytic activity (86.8% glycerol conversion and 61.5% 1,3-PDO yield). The results of in situ characterizations indicated that isolated tetragonal WO_4_ was formed and showed Lewis acidity, while the reaction of WO_4_ with spillover H atoms (from Pt metal) in situ generated Bronsted acid sites, in the reaction of glycerol and H_2_ over Pt/WO_x_-SBA-15 catalysts. The researchers considered that H-WO_4_ acted as the active site for the dehydration of glycerol (Figure 11), while metallic Pt had a role in H spillover, and the matching of the dimensions of isolated WO_4_ species and Pt nanoparticles was necessary for the selective hydrogenolysis of glycerol to 1,3-PDO.

**Figure 12:**
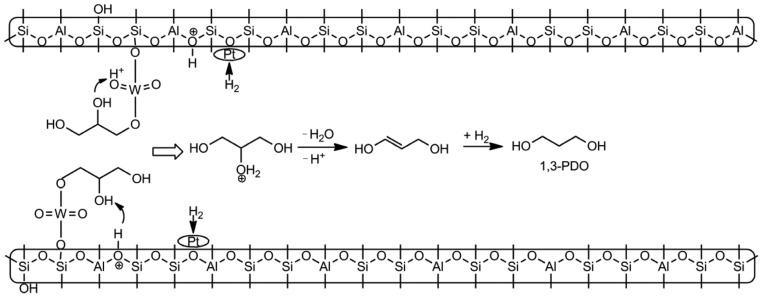
Plausible process for glycerol hydrogenolysis to 1,3-PDO on Pt/(W+Al)-SBA-15. Reproduced with permission from reference [80].

In addition, doping W and Al or other metal oxides could also modify the acidic properties of SBA-15 [80,81]. Feng et al. modified SBA-15 by doping W and Al with a hydrothermal process, and the obtained (W+Al)-SBA-15 had a uniform distribution of Brønsted acid and Lewis acid sites. The Pt/(W+Al)-SBA-15 catalyst displayed much higher activity for the hydrogenolysis of glycerol to 1,3-PDO than Pt/SBA-15 [80]. This benefitted from the synergistic effect between Bronsted acid and Lewis acid sites on the surface of Pt/(W+Al)-SBA-15, and this kind of interaction was relevant to the incorporation of W and Al species into the modified SBA-15 (Figure 12).

The pore structure of the SiO_2_ support, such as porous silica nanospheres and siliceous mesocellular foams (SiMCFs), can also have a great impact on the performances of the Pt/W-SiO_2_ catalysts [82,83]. Cheng et al. prepared W-doped SiMCF supports and Pt catalysts (Pt/W-SiMCFs) to investigate the influence of the structures of support W-SiMCFs on the catalytic performances in the hydrogenolysis of glycerol to 1,3-PDO [83].

Some zeolites such as SAPO-34 and H-MOR can provide acidic properties. Shi et al. [84] reported that the Pt/SAPO-34 catalyst showed good activity for the hydrogenolysis of glycerol but low selectivity for 1,3-PDO. They introduced WO_x_ into Pt/SAPO-34 and found that the doping amount of WO_x_ was important for the hydrogenolysis reaction. The suitable doping amount of WO_x_ was 20%, and the 20% WO_x_-Pt/SAPO-34 catalyst showed high activity and 1,3-PDO selectivity. They confirmed that the doping of WO_x_ to Pt/SAPO-34 increased the strengths of weak and moderate acids on the surface of WO_x_-Pt/SAPO-34, and uniformly dispersed WO_x_ species on WO_x_-Pt/SAPO-34 had strong interactions with Pt and SAPO-34 [84]. On the other hand, Pt catalysts supported on mordenite zeolite (H-MOR) without doped WO_x_ also showed good catalytic performances (94.9% glycerol conversion and 48.6% 1,3-PDO selectivity), and it was suggested that the Bronsted acid sites on the surface of H-MOR had an essential role in increasing the selectivity of 1,3-PDO [85].

**Figure 13:**
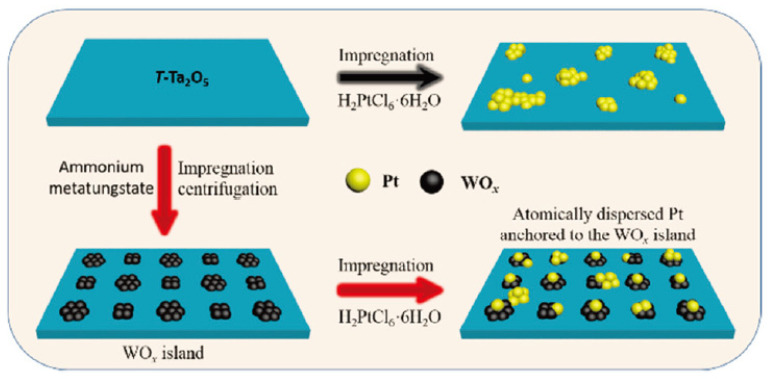
Illustration of preparation process of Pt^δ+^/WO_x_/T-Ta_2_O_5_. Reproduced with permission from reference [86].

Tantalum oxide (Ta_2_O_5_) is a characteristic oxide with outstanding thermal and hydrothermal stability, and it has almost no solid acidity. Ta_2_O_5_ was also used as the support of WO_x_ to enhance the dispersion of WO_x_ on the surface of Ta_2_O_5_ [86]. Zhao et al. prepared crystalline T-phase Ta_2_O_5_ via a solvothermal process, following calcination at 900 °C, and then loaded WO_x_ species on the surface of T-phase Ta_2_O_5_ via an impregnation process, and finally dispersed Pt onto WO_x_/T-Ta_2_O_5_ with a second impregnation step (Figure 13). The synthesized Pt^δ+^/WO_x_/T-Ta_2_O_5_ catalysts were applied to the hydrogenolysis of glycerol [86]. The researchers found that doping WO_x_ onto T-Ta_2_O_5_ was favorable for the dispersion of Pt at the atomic level, while Pt^δ+^ species that were stabilized by WO_x_ facilitated the adsorption and heterolytic dissociation of hydrogen, which was favorable for remarkably enhancing the in situ generation of Bronsted acid sites and the catalytic activity. The reaction results showed that Pt^δ+^/WO_x_/T-Ta_2_O_5_ with an extremely low content of WO_x_ exhibited remarkable efficiency of 1,3-PDO formation in the hydrogenolysis of glycerol, and this was ascribed to the good dispersion of Pt^δ+^ species and the synergistic effect of Pt and WO_x_/T-Ta_2_O_5_ [86].

TiO_2_ is also a commonly used support for loading Pt metal. For the Pt-WO_x_/TiO_2_ catalyst, the effect of the polymorph of TiO_2_ (rutile and anatase) on the catalyst structure and catalytic performances in the hydrogenolysis of glycerol to 1,3-PDO was investigated [87]. Zeng et al. reported that Pt-WO_x_/r-TiO_2_ showed good catalytic performances in the reaction, with a 1,3-PDO yield 38 times that of Pt-WO_x_/a-TiO_2_. The authors considered that this might be attributed to the structure and property differences of r-TiO_2_ and a-TiO_2_, with polymorph r-TiO_2_ facilitating faster H spillover from Pt nanoparticles to WO_x_ species than polymorph a-TiO_2_ [87].

#### 3.1.2. Doping and Modification of Pt Catalysts

Doping supports or modifying WO_x_ with other components is also one of the strategies to improve the performances of Pt-WO_x_ catalysts. Some researchers have also further investigated the doping effects on WO_x_/ZrO_2_ and WO_x_, such as MgO-doped WO_x_/ZrO_2_ [73], Li_2_B_4_O_7_-modified WO_x_/ZrO_2_ [74], sulfide-doped WO_x_/TiO_2_ [88], Nb-doped WO_x_ [89] and TiO_2_-doped WO_3_-ZrO_2_ [90]. For example, introducing Mg to Pt/WO_x_-ZrO_2_ could reduce WO_x_ polymerization on the surface of Pt/WO_x_-ZrO_2_ and decrease the amount of strong acids, while WO_x_ species with smaller sizes were favorable for the production of 1,3-PDO [73]. On the other hand, WO_x_ could interact with H^+^ to form H_2_WO_4_ during the hydrogenolysis reaction, and H_2_WO_4_ could be dissolved into the solution under reaction conditions, which would result in the deactivation of the Pt/WO_x_-ZrO_2_ catalyst.

However, modifying Pt/WO_x_-ZrO_2_ with Mg or Li_2_B_4_O_7_ could improve the hydrothermal stability of WO_x_-ZrO_2_, successfully prevent the leaching of WO_x_ species during the reaction, and significantly enhance the stability of the catalyst in the hydrogenolysis of glycerol [73,74]. Similarly, sulfate doping into the TiO_2_-supported Pt-WO_x_ catalyst also improved the catalytic stability of Pt/WO_x_-SO_4_^2−^/TiO_2_ in the hydrogenolysis of glycerol, due to the leaching of Pt being greatly inhibited and due to the modification of WO_x_ with sulfates [88]. In addition, the Pt/WO_x_ catalyst had the problem of over-reduction of WO_x_ species under hydrogen pressures during the hydrogenolysis reaction [89]. To solve this problem, WO_x_ was modified. Yang et al. reported that doping Nb into Pt/WO_x_ could prevent the over-reduction of WO_x_ support during the hydrogenolysis of glycerol, considerably widen the optimal H_2_ pressure from 1.0 to 5.0 MPa, and ensure a relatively high stability of the Pt catalyst during the long reaction time [89].

There were also some studies on the modification of Pt/WO_x_ catalysts with precious metals, such as Au or Ru. Wang’s group investigated the promoting effect of Au on the catalytic performances of Pt/WO_3_ [91,92] and Pt/WO_x_/Al_2_O_3_ [93] in the hydrogenolysis of glycerol to 1,3-PDO. Zhao et al. first prepared Pt/WO_x_ by impregnating WO_x_ with H_2_PtCl_6_ solution, and then they deposited Au onto the surface of Pt/WO_x_ by using HAuCl_4_ and NaBH_4_ [91]. They applied the Au/Pt/WO_x_ catalysts in the hydrogenolysis of glycerol and investigated the influence of introducing Au to Pt/WO_x_ on the acidic properties of the catalysts and the selectivity to 1,3-PD. The results showed that doping Au onto Pt/WO_x_ could promote the production of frustrated Lewis pairs, thus decreasing the original Lewis acid sites on the surface of Pt/WO_x_ but increasing the in situ generation of Brønsted acid sites in a hydrogen atmosphere; consequently, the in situ generated H^+^ and H^−^ pairs separated and served as the active sites in the hydrogenolysis of glycerol to 1,3-PD [91].

**Figure 14:**
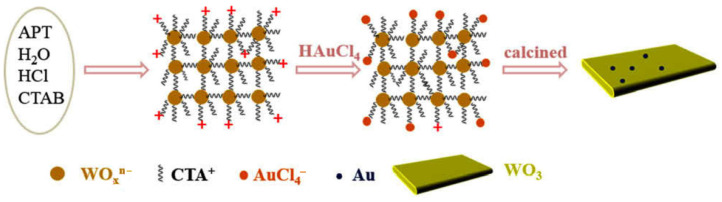
A new CTAB-assisted deposition approach to prepare Au/WO_3_. Reproduced with permission from reference [92].

Yang et al. also investigated Pt/Au/WO_3_ catalysts to understand the promotional effect of gold [92]. They first prepared Au/WO_3_ via a CTAB-assisted surface-modified deposition process (using (NH_4_)_10_W_12_O_41_·5H_2_O, HCl, CTAB, and HAuCl_4_ as the starting materials), and then they deposited Pt on the surface of Au/WO_3_ via the impregnation method (Figure 14). The research results showed that doping Au caused partial replacement of W^6+^ by Au^3+^, weakened the interaction between Pt and WO_3_, enhanced the low-temperature reduction of Pt and W, increased the uniform dispersion of Pt species on the surface of WO_3_, and consequently led to improved catalytic performances of Pt/Au/WO_3_ catalysts (with W and Pt in lower valence) [92]. They indicated that these changes in the electronic properties were favorable for converting glycerol to 1,3-PDO.

In addition, Wang et al. [93] further doped gold into the parent Pt/WO_x_/Al_2_O_3_ to study the interaction between Au and Pt/WO_x_/Al_2_O_3_ as well as the effect on the catalytic performances. The reaction and characterization results revealed that the electron transfer from W to Au weakened the strong metal support interaction between Pt and WO_x_, enlarged the exposed Pt surface and increased the H-spillover capacity as well as the number of interfacial sites, therefore increasing the yield of 1,3-PDO.

Similarly, the modification of the Pt/WO_x_ catalysts with Ru was also investigated. Wen et al. [94,95] reported that doping Ru facilitated the reduction of Pt at lower temperatures, restrained the sintering of metal particles on Pt-Ru/WO_x_/Al_2_O_3_, promoted the W^6+^ ↔ W^5+^ cycle, enhanced the capability for H_2_ adsorption, and increased the number of Bronsted acid sites on the surface of Pt-Ru/WO_x_/Al_2_O_3_. The catalyst of 2Pt-1Ru/WO_x_/Al_2_O_3_ showed high activity for the hydrogenolysis of glycerol to 1,3-PDO compared to 2Pt/WO_x_/Al_2_O_3_ [94]. Meanwhile, the 2Pt-1Ru/WO_x_/Al_2_O_3_ catalyst exhibited more stability in the hydrogenolysis of glycerol than the 2Pt/WO_x_/Al_2_O_3_ catalyst because the active sites on 2Pt/WO_x_/Al_2_O_3_ easily agglomerated, resulting in the deactivation of 2Pt/WO_x_/Al_2_O_3_, while the Pt-Ru active sites on the 2Pt-1Ru/WO_x_/Al_2_O_3_ catalyst could be stabilized via the addition of Ru [95].

#### 3.1.3. Effects of the Structures and Properties of WO_x_ and Supports

**Figure 15:**
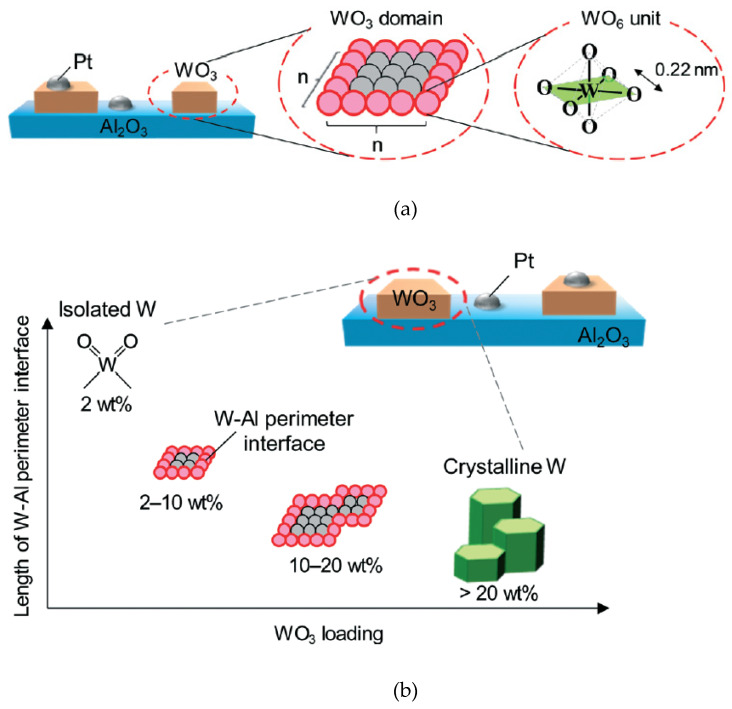
**(a)** Structural models of a WO_3_ monolayer domain and (**b**) W species on Pt/WO_3_/Al_2_O_3_ catalysts. Reproduced with permission from reference [96].

**Figure 16:**
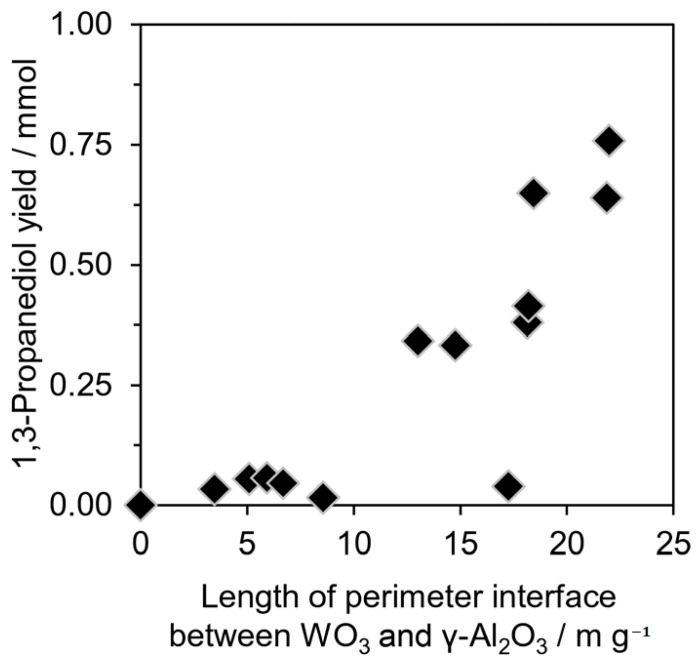
Effect of the perimeter interface length between WO_3_ and γ-Al_2_O_3_ on hydrogenolysis over Pt/WO_3_/Al_2_O_3_ catalysts. Reproduced with permission from reference [96].

The morphologies and structures of WO_x_ and supports will also have a great impact on the performances of Pt/WO_x_-based catalysts. For Pt/WO_3_/Al_2_O_3_, Aihara et al. [96] investigated the influence of the length of the perimeter interface between the monolayer domain of two-dimensional WO_3_ (2D WO_3_) and γ-Al_2_O_3_ on the catalytic activity for the selective hydrogenolysis of glycerol. The characterizations with XRD, XPS, and XAFS techniques revealed that, in the range of WO_3_ content below 20 wt%, the monolayer domains of 2D WO_3_ were formed on the surface of γ-Al_2_O_3_. On the other hand, the H_2_-TPR results showed the existence of two types of W species, which were loaded on the surface of γ-Al_2_O_3_ and had dissimilar reduction properties. That is, W species inside the WO_3_ domain were not easily reduced compared to the W species at the edge of a WO_3_ domain (Figure 15) [96]. The authors indicated that the formation of 1,3-PDO in the hydrogenolysis of glycerol was positively correlated with the length of the W-Al peripheral interface (Figure 16), and they suggested that a W-(OH)-Al site acted as the main active site, which was located at the W-Al perimeter interface.

On the other hand, Xu et al. investigated the influence of the pore structure of γ-Al_2_O_3_ when used as the support of Pt-WO_x_ on the activities of the catalysts for the hydrogenolysis of glycerol in a flow-type fixed bed reactor [97]. The three kinds of aluminum oxide supports were prepared via a hydrothermal crystallization process, and these Al_2_O_3_ supports had different pore structures, that is, rod-like, flake-like, and spindle-like (named Al_2_O_3_-R, Al_2_O_3_-F, and Al_2_O_3_-SP, respectively), but the contents of Pt and WO_x_ were regulated to remain the same on these different alumina supports. The research results showed that the flake-like Pt/WO_3_/Al_2_O_3_-F catalyst had an open pore structure with 2D (two-dimensional) nanosheets, while rod-like Pt-WO_x_/Al_2_O_3_-R had a large specific surface area. Compared to rod-like Pt-WO_x_/Al_2_O_3_-R, flake-like Pt/WO_3_/Al_2_O_3_-F remarkably enhanced the accessibility of the active sites on the pores of the catalyst, consequently increasing the catalytic activity and showing higher selectivity for the formation of 1,3-PDO (38.2% yield) under high space velocity and low Pt content [97].

**Figure 17:**
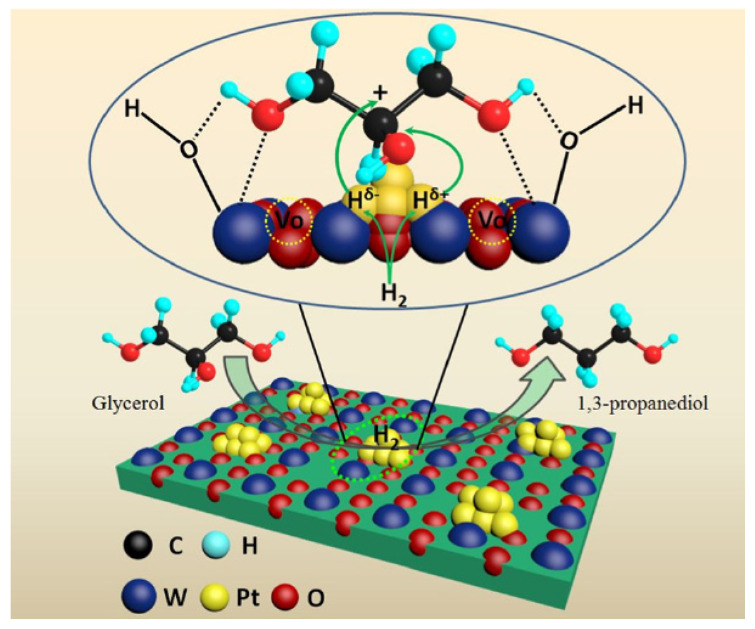
Proposed reaction mechanism for glycerol hydrogenolysis to 1,3-PDO over the Pt/H-WO_3_ catalyst. Reproduced with permission from reference [98].

The role of oxygen defects in enhancing the formation of 1,3-PDO over Pt/WO_x_ catalysts was investigated [98,99]. Niu et al. [98] prepared WO_3_·0.33H_2_O with different contents of oxygen vacancies (VO) and employed them as the support of Pt for the hydrogenolysis of glycerol. The results showed that the oxygen vacancies of WO_3_ greatly affected Pt metal dispersion, the surface acidity of the catalysts, and the interaction between WO_3_ and Pt. The Pt catalyst supported on WO_3_, having a high oxygen vacancy (named Pt/H-WO_3_), revealed considerably high activity and selectivity for 1,3-PDO, compared to the catalyst Pt/L-WO_3_, which had low oxygen vacancies (L-WO_3_). This was considered to be due to the roles of surface Bronsted acid sites on the Pt/H-WO_3_ catalyst and the synergistic effect of WO_x_ species and Pt metal [98]. They proposed a possible mechanism (Figure 17) for the hydrogenolysis of glycerol to 1,3-PDO over Pt/H-WO_3_, in which glycerol adsorbed on the surface of the catalyst and the two terminal hydroxy groups coordinated with the unsaturated W ions, while the secondary hydroxy group reacted with the Bronsted acid site from Pt-(WO_x_)_n_-H (H^δ+^ species generated in situ) [98].

**Figure 18:**
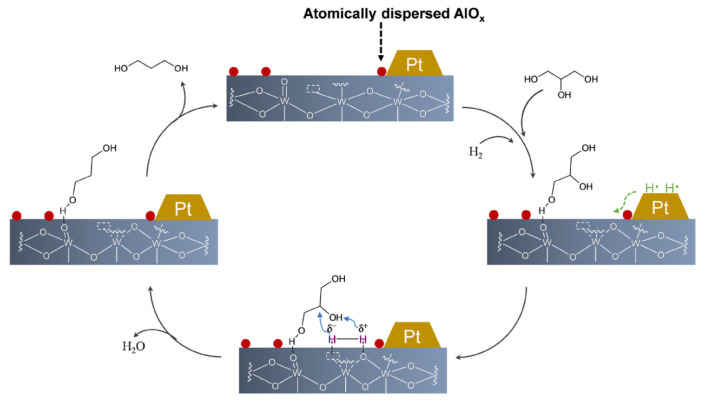
Proposed mechanism of glycerol hydrogenolysis to 1,3-PDO on the surface of Pt/WO_x_ modified with atomically dispersed AlO_x_. Reproduced with permission from reference [99].

Yang et al. also investigated the regulating oxygen defects for enhancing the hydrogenolysis of glycerol by atomically dispersing alumina on a Pt/WO_x_ catalyst; the results showed that highly dispersed alumina could promote the formation of oxygen vacancies in WO_x_ in situ in a hydrogen atmosphere, which could increase the ability of the Pt/Al-WO_x_ catalyst to adsorb glycerol and enhance the capability of Bronsted acid sites to activate the secondary C–O bond, thus significantly increasing the catalytic performances in the hydrogenolysis of glycerol [99]. They concluded that introducing highly dispersed AlO_x_ could weaken the strong metal−support interactions between WO_x_ species and Pt; thus, more Pt species were exposed, which facilitated the in situ generation of oxygen vacancies in WO_x_ near Pt species. The authors also deduced a possible reaction mechanism, as shown in Figure 18, wherein the primary -OH of glycerol adsorbed on the surface oxygen defects (Lewis acid sites), and hydrogen atoms spilled over from the Pt metal surface onto WO_x_ and reduced WO_x_ to form Bronsted acid sites [99].

Ma’s group employed a kind of inert SiO_2_ as the support of Pt-(WO_x_)_n_-H, and prepared Pt-(WO_x_)_n_-H/SiO_2_ model catalysts, utilizing the method of metal oxide interaction [100]. The authors explored the nature of the Bronsted acid site of Pt-(WO_x_)_n_-H, investigated the influence of WO_x_ structure and reduction degree on the catalytic performances, and probed the atom efficiency of W species to the formation of 1,3-PDO in glycerol hydrogenolysis [100]. The research results revealed that both the reduction degree and polymerization of WO_x_ could affect the Bronsted acidity of Pt-(WO_x_)_n_-H, and the WO_x_ domains with medium polymerization and without reduction showed the strongest Bronsted acidity and the lowest apparent activation energy in the hydrogenolysis of glycerol. On the other hand, compared with isolated HWO_4_ species, the clusters of dimeric HW_2_O_7_ delocalized more negative charge to the broader surface of Pt nanoparticles, resulting in a much stronger Bronsted acidity. Therefore, the hydrogenolysis of glycerol to 1,3-PDO effectively occurred only when the strong Bronsted acid site and activated hydrogen coexisted [100].

Li et al. prepared a series of supported Pt-WO_x_ catalysts (Pt-WO_x_/TiO_2_, Pt-WO_x_/ZrO_2_, and Pt-WO_x_/Al_2_O_3_) and investigated the effect of the surface density of WO_3_ on the catalytic performances of the catalysts in the selective hydrogenation of glycerol to 1,3-PDO [101]. The results showed that the catalytic activity and selectivity for 1,3-PDO sensitively depended on the WO_3_ surface density. They found that the dispersion of WO_x_ species was a key factor affecting the performances of Pt-WO_x_ catalysts, and the WO_x_ species uniformly dispersed on the surface (with surface densities of 1.5–2.0 W/nm^2^) showed much higher activity in the formation of 1,3-PDO. The synergistic effect and electron transfer between the WO_x_ domains and Pt particles were also confirmed [101].

Dolsiririttigul et al. [102] prepared Pt-WO_x_/Al_2_O_3_ catalysts with different contents of WO_x_ and pretreatment conditions, and they employed them in the hydrogenation of glycerol to investigate the relationships between the catalyst structures and catalytic performances of Pt-WO_x_/Al_2_O_3_. They found that increasing the content of WO_x_ could improve the uniform dispersion of Pt particles, showing an interaction between Pt metal and WO_x_, and W-O-W clusters were created at an optimum W loading content (15%), which was conducive to the stabilization of H spillover from the surface of Pt metal via the delocalization of electrons. On the other hand, the calcination temperatures of WO_x_/Al_2_O_3_ influenced the dispersion of WO_x_ species over the Al_2_O_3_ surface, and calcining WO_x_/Al_2_O_3_ at 800 °C could make isolated WO_x_ species connect to form W-O-W clusters, which were related to the maximum catalytic performances of Pt-WO_x_/Al_2_O_3_ [102].

### 3.2. Ir-Based Catalysts

In addition to the Pt-WO_x_ catalytic system, Ir-ReO_x_ and Rh-ReO_x_ are another group of catalytic systems applied in glycerol hydrogenolysis that have received great attention from researchers. The Tomishige group first reported the catalytic performances of Ir-ReO_x_ and Rh-ReO_x_ catalysts in the hydrogenolysis of glycerol to 1,3-PDO [64,65,66]. Subsequently, multiple research groups conducted further investigations on the catalytic performances and reaction mechanism of Ir-ReO_x_ and Rh-ReO_x_ catalysts in the hydrogenolysis of glycerol, and relevant reports can be found in review papers [20,103]. Although a single-component Ir catalyst (Ir/SiO_2_) showed almost no activity in the hydrogenolysis of glycerol compared to a single-component Rh catalyst (Rh/SiO_2_), the combination of Ir and Re (Ir-ReO_x_-based catalysts) revealed higher activity than Rh-ReO_x_-based catalysts [103]. Here, several works [104,105,106,107,108,109] on Ir-based catalysts for the selective hydrogenolysis of glycerol to 1,3-PDO are reviewed.

Ir-ReO_x_ is usually a supported catalyst, and the Tomishige group initially investigated ReO_x_-modified Ir nanoparticles supported on SiO_4_ with 4 wt% Ir loading (Re/Ir = 1) [64,65]. However, the hydrogenolysis of glycerol over Ir-ReO_x_/SiO_2_ (with 4 wt% Ir loading) usually requires the addition of a small amount of sulfuric acid to stabilize the catalytic active sites [65]. Liu et al. [104] further investigated the selective hydrogenolysis of glycerol over an Ir-ReO_x_/SiO_2_ catalyst with a higher Ir loading amount (20 wt%) but without sulfuric acid addition, and they found that 20 wt% Ir-ReO_x_/SiO_2_ (no sulfuric acid addition) had comparable activity to the 4 wt% Ir-ReO_x_/SiO_2_ + H_2_SO_4_ catalytic system (in previous report [65]), while maintaining high 1,3-PDO selectivity. The detailed characterization results indicated that after the reaction over the catalysts, the reduction degree of Re species on the surface of 20 wt% Ir-ReO_x_/SiO_2_ was higher than that on the catalyst of 4 wt% Ir-ReO_x_/SiO_2_.

Additionally, the reusability of 20 wt% Ir-ReO_x_/SiO_2_ without sulfuric acid addition was confirmed under optimized operation conditions [104]. Furthermore, Liu et al. [105] employed anatase-TiO_2_, rutile-TiO_2_, P25-TiO_2_, ZrO_2_, CeO_2_, γ-Al_2_O_3_, MgO, activated carbon, and SiO_2_ as the supports of Ir-ReO_x_, and they investigated the influence of supports on the catalytic performances of Ir-ReO_x_/support (without the addition of sulfuric acid) in the hydrogenolysis of glycerol. They found that the rutile-TiO_2_ support showed high activity, while natase-TiO_2_, ZrO_2_, CeO_2_, Al_2_O_3_, MgO, SiO_2_, and activated carbon supports showed very low activity. The characterization and reaction results also revealed a relationship between catalyst structures and the catalytic activity of Ir-ReO_x_/rutile-TiO_2_. The authors indicated that there was an interaction between the partially oxidized ReO_x_ cluster and Ir metal particles, the Ir-ReO_x_ interface served as the active site, and the uniformly dispersed small Ir-ReO_x_ particles could be stabilized by the rutile-TiO_2_ support [105].

For the Ir-ReO_x_/SiO_2_ (4 wt% Ir) + H_2_SO_4_ catalyst system [64], the presence of sulfuric acid was not beneficial for the reactor and catalyst circulation, and therefore, researchers tried to use solid acids, such as ion-exchange resins, silica–alumina, and zeolites, instead of sulfuric acid as additives for the hydrogenolysis of glycerol to 1,3-PDO [106]. In addition, some solid acids were also used as the support of Ir-ReO_x_. For example, Chanklang et al. [107] employed H-ZSM-5 as the support of Ir-ReO_x_ and investigated the catalytic performances of Ir-ReO_x_/H-ZSM-5 for the hydrogenolysis of glycerol to 1,3-PDO without adding acidic additives, and the synergistic effect between Re and Ir on the support of H-ZSM-5. They found that Ir-ReO_x_/H-ZSM-5 (with 4 wt% Ir loading and Re/Ir = 1) showed high activity in the hydrogenolysis of glycerol, Ir species were uniformly dispersed on the H-ZSM-5 surface in the presence of ReO_x_, and there was an electronic interaction between Ir and Re over Ir-ReO_x_, resulting in the enhancement of catalytic activity and selectivity for the hydrogenolysis of glycerol to 1,3-PDO [107].

**Figure 19:**
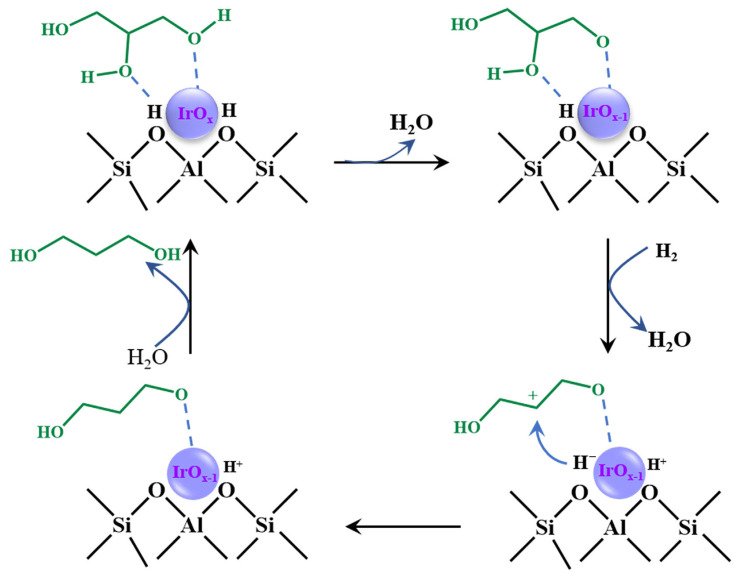
Proposed reaction mechanism for glycerol hydrogenolysis to 1,3-PDO over the IrOx/H-ZSM-5 catalysts. Reproduced with permission from reference [108].

On the other hand, researchers have also tried to only use solid acids as the support of Ir metal without the addition of ReO_x_ as the co-catalyst. For example, Wang et al. [108] prepared a monocomponent iridium catalyst supported on H-ZSM-5 solid acid but without using Re oxophilic metal oxide to modify the catalyst, and they applied it for the hydrogenolysis of glycerol to 1,3-PDO but with no addition of acid additives. The results showed that high glycerol conversion and selectivity for 1,3-PDO could be achieved over IrOx/H-ZSM-5 under optimized reaction conditions. Ir-induced Bronsted acid sites—that is, Ir-O(H)-H-ZSM-5—could be the active sites of the IrOx/H-ZSM-5 catalyst, and the ratio of Bronsted acid sites/overall acid sites could greatly affect the catalytic activity and 1,3-PDO selectivity. The authors speculated that at the interface between IrOx and H-ZSM-5, via H spillover and reversed H spillover, Ir-O(H)-H-ZSM-5 sites were formed through the synergistic interaction between H-ZSM-5 and IrOx, and they proposed a plausible mechanism to clarify the role of Ir-O(H)-H-ZSM-5 sites in the hydrogenolysis of glycerol (Figure 19) [108].

For Ir-ReO_x_-catalyzed glycerol hydrogenolysis, the working mechanism of the addition of solid and liquid acids -n hydrogenolysis seems slightly different. Via experimental and theoretical investigations, Zhou et al. [109] reported a reaction mechanism for acid-affected hydrogenolysis of glycerol to 1,3-PDO over Ir-Re/SiO_2_ catalysts modified with solid acids and inorganic liquid acids (HZSM-5, Amberlyst-15, H_2_SO_4_, HNO_3_, HCl) to explain the acid effects on the reaction. The authors identified the reaction pathway and rate-determining step (RSD) of glycerol hydrogenolysis to 1,3-PD on the Ir-Re surface via density functional theory calculations and further investigated the origin of the catalytic performances of Ir-Re enhanced by acids. They elucidated that the significant enhancement of glycerol hydrolysis by solid acids could be attributed to the diminishing of the energy barrier of the rate-determining step due to the H proton. However, in the cases of H_2_SO_4_, HNO_3_, and HCl, these inorganic liquid acids might cause some negative effects on the catalytic performances due to the anion adsorption of the liquid acids [109].

## 4. Conclusions and Outlook

For the hydrogenolysis of glycerol to 1,2-PDO, nonprecious metals are mainly used as the active components of catalysts. From the perspective of the separation of catalysts and products, as well as catalyst recycling, solid catalysts, especially supported metal catalysts, are widely employed. The nonprecious metals used as active components on supported catalysts mainly include Cu, Ni, Co, etc. Among them, the catalysts with Cu as the active component have the best catalytic activity and show high selectivity for 1,2-PDO. The size of the Cu nanoparticles affects the activity of the Cu-based catalysts, and small Cu nanoparticles favor high catalytic activity for the selective hydrogenolysis of glycerol to 1,2-PDO.

The catalysts for glycerol hydrogenolysis usually have bifunctional active sites (hydrogenation and dehydration sites) because the reaction involves dehydration and hydrogenation steps. Usually, acid supports or base supports are used to construct dehydration active sites. γ-Al_2_O_3_, acidic zeolites (for example, H-β, H-Y, H-MOR, H-ZSM-5, etc.), and other acidic metal oxides or mixed oxides are often used as acid supports, while MgO, La2O3, alkali-modified metal oxides, and hydrotalcites are often used as base supports. Al_2_O_3_ is the most widely investigated acidic support of Cu-based catalysts in this research area, and Cu/MgO with basic sites is also highly active, selective, and stable for the hydrogenolysis of glycerol to 1,2-PDO. On the other hand, Cu-Al-Zn catalysts have both basic and acidic sites, which respond to catalytic hydrogenation and impact the cleavages of the C–C bond and C–O bond of glycerol.

For the hydrogenolysis of glycerol to 1,3-PDO, precious metals, such as Pt, Ru, Pd, Rh, and Ir, are mainly adopted as the active components of bifunctional catalysts, and furthermore, the requirements for the properties of dehydration active sites (acid sites) and hydrogenation active sites (metal sites) on the catalysts are not the same as those for the production of 1,2-PDO. The Bronsted acid sites on the surface of bifunctional catalysts and the synergistic interaction between Bronsted acid sites and metal active sites are the key factors for the generation of 1,3-PDO, and modifying these precious metal catalysts with some oxygenate metal oxides, such as WO_x_, MoO_x_, and ReO_x_, could produce Bronsted acid sites in situ in hydrogenolysis. Among them, Pt metal modified with WO_x_ (Pt-WO_x_-based catalysts) and Ir metal modified with ReO_x_ (Ir-ReO_x_-based catalysts) show high catalytic activities and selectivity for 1,3-PDO. Pt-WO_x_ or Ir-ReO_x_ is usually supported on thermally stable materials with large specific surface areas. Some metal oxides, such as Al_2_O_3_, ZrO_2_, TiO_2_, SiO_2_, and their composite oxides, as well as some zeolites (SAPO-34 and H-MOR) are commonly employed as supports.

Using suitable supports can enhance the interaction between W species and supports, which is beneficial for the dispersion of WO_x_ species and Pt nanoparticles and, therefore, promotes the formation of Pt-WO_x_ interfaces. For Pt-WO_x_-based catalysts, isolated Pt nanoparticles contribute to the H_2_ dissociation capacity, while WO_x_ provides specific Bronsted acid sites and is involved in dehydration–hydrogenation reactions. The Bronsted acid sites are produced in situ through hydrogen dissociation on Pt metal and spillover to WO_x_, and glycerol hydrogenolysis occurs at the Pt-WO_x_ interface. Pt particle size, WO_x_ domain size, oxygen vacancies, the surface area of WO_x_, and the interaction between WO_x_ and Pt particles play important roles in the hydrogenolysis of glycerol to 1,3-PDO. The abundant oxygen vacancies and large surface area of WO_x_ can improve the Pt dispersion and stabilize the Pt isolation. The hydrogenolysis of glycerol is structurally sensitive to Pt particle size and WO_x_ domain size, and medium polymerized WO_x_ is beneficial for the formation of 1,3-PDO.

Ir-ReO_x_-based catalysts show unique behaviors in glycerol hydrogenolysis. The addition of sulfuric acid into the reaction system of glycerol hydrogenolysis catalyzed by Ir-ReO_x_/SiO_2_ is not desirable because the presence of sulfuric acid is not beneficial for either the reactor or catalyst circulation. Therefore, employing solid acids as additives or directly as supports for Ir-ReO_x_ is ideal. Using an acidic zeolite (such as H-ZSM-5) or rutile-TiO_2_ as the support of Ir-ReO_x_ has positive effects on the catalytic activity of Ir-ReO_x_ for the hydrogenolysis of glycerol to 1,3-PDO, and these supports can afford a distinctive circumstance for the stabilization of Ir-ReO_x_ particles in which the interaction between Ir and ReO_x_ is enhanced, and accordingly, the conversion of glycerol and the formation of 1,3-PDO are promoted.

Looking ahead to future research, for the hydrogenolysis of glycerol to 1,2-PDO, further focus should be directed toward the stability of Cu-based catalysts, to address the issue of catalyst deactivation due to sintering or carbon deposition. The stability of Cu-based catalysts is crucial for the commercial application of glycerol hydrogenolysis to 1,2-PDO. It is expected that research on the use of composite supports, improvement of support structures, and enhancement of Cu and support interactions will be strengthened to enhance the stability of Cu-based catalysts. In addition, detailed investigations on the catalytic mechanism of Cu-based catalysts for the hydrogenolysis of glycerol to 1,2-PDO are also needed, which can provide feedback to regulate the structures and properties of Cu-based catalysts, thereby improving the performances of the catalysts.

For the hydrogenolysis of glycerol to 1,3-PDO, most research results currently indicate that Pt-WO_x_-based and Ir-ReO_x_-based catalysts are more effective than other catalysts for the hydrogenolysis of glycerol to 1,3-PDO. The catalysts for the conversion of glycerol to 1,3-PDO typically require special structures and surface interfaces (such as the surface properties possessed by Pt-WO_x_ or Ir-ReO_x_) due to the selective activation of the secondary -OH group of glycerol. However, the performances of Pt-WO_x_-based and Ir-ReO_x_-based catalysts still need further improvement. The precise control of the structures and surface properties of Pt-WO_x_ and Ir-ReO_x_ is very important. The domain size of WO_x_ or ReO_x_ and the interaction between the metal (Pt or Ir) and the oxide (WO_x_ or ReO_x_) have significant impacts on the formation of 1,3-PDO. Therefore, investigations on these aspects are promising. In addition to the Bronsted acid sites, the influence of Lewis acid sites, as well as the equilibrium ratio of Bronsted acid and Lewis acid in glycerol adsorption and the dehydration step, also should be paid attention. Elsewhere, the stability of Pt-WO_x_ and Ir-ReO_x_ catalysts could also be a focus of future research. Additionally, the aggregation of Pt or Ir metal particles, the leaching of WO_x_ and ReO_x_ in the reaction, and the influence of hydrothermal environments on catalysts need to be considered. Furthermore, the detailed mechanisms of glycerol hydrogenolysis over Pt-WO_x_ and Ir-ReO_x_ catalysts are to be further studied. Plus, in future research, the combination of quantum chemistry calculations with in situ characterizations of the catalysts is desirable. Lastly, Pt and Ir are both precious metals, and future research will hopefully lead to the development of nonnoble metal catalysts to replace these precious metals.

## Figures and Tables

**Table 1:** Supported Cu-based catalysts for glycerol hydrogenolysis to 1,2-PDO. Reproduced with permission from reference [25].

Catalyst	Reaction Conditions				Selectivity (%)		
	Temp. (°C)	Press. (bar)	T (h)	Conv. (%)	1,2-PDO	Acetol	EG
RANEY Cu	240	30	-	86	66	-	-
CuO/ZnO	180	80	90	20	100		
Cu:ZnO	200	42	12	17	29	-	3
Cu–ZnO (1:1)	200	60	6	2.6 TOF	94	-	6
CuO/ZnO–OG ^a^	200	70	7	46	90	1	1
CuO/ZnO–CP ^b^				17	87	1	0
Cu_1.1_/ZnO–HKUST-1 ^m^	230	20	2	>99	95.6	4.4	-
20% Cu/ZnO/PAA ^n^ 2_0-NC_ (not calcined)	200	30	24	70	87	-	12
20% Cu/ZnO/PAA5_20-NC_	200	30	24	70	87	-	13
Cu/Al_2_O_3_, HY, HZSM, Hβ	200	36	10	35	93.9	-	-
Cu–STA/Al_2_O_3_	240	60	-	90	90	-	4
Cu/Al_2_O_3_	200	50	24	59	79	7	1
Cu–Al	220	40	-	60	90	5	3
Cu–Al CAP(Na_2_CO_3_)	220	52	5	62	88	1	10
Cu/fly ash	220	52	5	37	84	4	3
CuAl_2_O_4_	220	50	12	90	83	-	-
Cu/boehmite	200	40	-	77	93	-	0.2
Cu/Al_2_O_3_	180–300	1		100	88	9	-
Cu/Al_2_O_3_	200	50	24	76	96	1	1
Cu/Al_2_O_3_	205	50	23	89	94	-	-
15% Cu/Al_2_O_3_	200	1	180 g cat min mol^−1^	100	60	30	-
60% Cu/Al_2_O_3_	250	~50	SLV ^o^ 0.55 (m^3^m^−2^ h^−1^)	90	91.5	-	1.5
Cu/SiO_2_ PG ^c^	200	90	12	73	94	Trace	2
Cu/SiO_2_ IM ^d^				26	95	0.3	2
Cu/SiO_2_-Na	180	90	12	33	98.7	Trace	0.7
Cu/SBA-15	250	83	6-7	96	92	3	3
Cu/SiO_2_ IW ^e^	245	240 (218)	-	13	76	-	-
Cu/SiO_2_ IE ^f^	255			100	87	5	4
Cu/SiO_2_ Hom-DP ^g^	200	90	12	55	96	-	2
Het-DP ^h^				65	93	-	7
3CuB/SiO_2_	200	50	150	100	98	0.5	0.5
10% Cu/SBA-15(G) ^i^	210	40	1.5	59	98.7	-	~1
10% Cu/SBA-15(IM)	210	40	1.5	8	99.2	-	0.8
CuO-15 MgO-CP;	180	30	20	72	98	-	1.3
CuO-15 MgO-CP + NaOH				82	96	-	0.3
Cu/MgO	200	40	8	49.3	92	-	6
Cu/Mg–Al mixed-oxide	210	90	24	>95	85	-	-
10% Cu/MgO	220	7.5	1.2^h−1^				
(WHSV) ^p^	100	95.5 (y)	1.6	-	105		
Cu_0.4_/Mg_5.6_Al_2_O_8.6_-CP (75% aq. gly)	180	30	20	80	98.2	0.0	1
Cu–Zn_10_HT_r_ ^j^	200	20	8	38.5	61.6	-	-
5% CuO/Mg_9_Al_2.7_–Ga_2.3_O_2_	220	40 mL min^−1^	23	95	97	-	-
Cu-Delaminated hectorites	200	40	8	61.4	93	-	-
20% Cu/Dolomite	180	20	6	100	92	6.2	-
20% Cu/Dolomite	200	40	10	78.5	79	19	-
7% Cu/CGran ^k^ (750)	220	50	-	22	94.7	3	-
15% Cu(M)–Zr(C) ^l^	240	20	24	80	95	-	-

^a^ OG—oxalate gel. ^b^ CP—co-precipitation. ^c^ PG—precipitation gel. ^d^ IM—Impregnation. ^e^ IW—incipient wetness. ^f^ IE—ion exchange. ^g^ Hom-DP—homogenous deposition. ^h^ Het-DP—heterogenous deposition. ^i^ G—grinding. ^j^ HTr—Hydrotalcite reduced. ^k^ CGran—Cu supported on activated carbon. ^l^ Cu(M)–Zr(C)-M: metal–organic-framework mediated synthesis route, C: commercial. ^m^ HKUST-1-[Cu_3_(BTC)2·9.4(H_2_O), BTC = benzene1,3,5-tricarboxylic acid]. ^n^ PAA—poly acrylic acid. ^o^ SLV—superficial liquid velocity. ^p^ WHSV—weight hourly space velocity.

**Table 2:** Sizes of Cu crystallites in Cu-Zn-Al catalysts and their performances in selective glycerol hydrogenolysis to 1,2-PDO. Reproduced with permission from reference [30].

Catalyst	Size of Cu Crystallites (nm)	Glycerol Conversion (120 min)	Selectivity to 1,2-Propanediol (120 min)
HTc350	15.7	63.1	0.96
HTc700	29.0	38.0	0.97
Hydrox	38.5	29.6	0.88
OXAL	17.0	27.6	0.93
Combus	42.4	20.0	0.92
Mixox	74.0	3.2	0.56

Testing at 230 °C and hydrogen pressure of 7.0 MPa, catalyst concentration of 2.4 wt%.

## Data Availability

Not applicable.
